# Unmanned aerial vehicle based multi-person detection via deep neural network models

**DOI:** 10.3389/fnbot.2025.1582995

**Published:** 2025-04-17

**Authors:** Mohammed Alshehri, Laiba Zahoor, Yahya AlQahtani, Abdulmonem Alshahrani, Dina Abdulaziz AlHammadi, Ahmad Jalal, Hui Liu

**Affiliations:** ^1^Department of Computer Science, King Khalid University, Abha, Saudi Arabia; ^2^Faculty of Computer Science, Air University, Islamabad, Pakistan; ^3^Department of Informatics and Computer Systems, King Khalid University, Abha, Saudi Arabia; ^4^Department of Information Systems, College of Computer and Information Sciences, Princess Nourah Bint Abdulrahman University, Riyadh, Saudi Arabia; ^5^Department of Computer Science and Engineering, College of Informatics, Korea University, Seoul, Republic of Korea; ^6^Cognitive Systems Lab, University of Bremen, Bremen, Germany; ^7^Guodian Nanjing Automation Company Ltd., Nanjing, China; ^8^Jiangsu Key Laboratory of Intelligent Medical Image Computing, School of Future Technology, Nanjing University of Information Science and Technology, Nanjing, China

**Keywords:** unmanned aerial vehicle, neural network models, deep learning, human action recognition, CNN, RNN, image processing, action classification neural network models

## Abstract

**Introduction:**

Understanding human actions in complex environments is crucial for advancing applications in areas such as surveillance, robotics, and autonomous systems. Identifying actions from UAV-recorded videos becomes more challenging as the task presents unique challenges, including motion blur, dynamic background, lighting variations, and varying viewpoints. The presented work develops a deep learning system that recognizes multi-person behaviors from data gathered by UAVs. The proposed system provides higher recognition accuracy while maintaining robustness along with dynamic environmental adaptability through the integration of different features and neural network models. The study supports the wider development of neural network systems utilized in complicated contexts while creating intelligent UAV applications utilizing neural networks.

**Method:**

The proposed study uses deep learning and feature extraction approaches to create a novel method to recognize various actions in UAV-recorded video. The proposed model improves identification capacities and system robustness by addressing motion dynamic problems and intricate environmental constraints, encouraging advancements in UAV-based neural network systems.

**Results:**

We proposed a deep learning-based framework with feature extraction approaches that may effectively increase the accuracy and robustness of multi-person action recognition in the challenging scenarios. Compared to the existing approaches, our system achieved 91.50% on MOD20 dataset and 89.71% on Okutama-Action. These results do, in fact, show how useful neural network-based methods are for managing the limitations of UAV-based application.

**Discussion:**

Results how that the proposed framework is indeed effective at multi-person action recognition under difficult UAV conditions.

## 1 Introduction

Human Action Recognition (HAR) has been gaining a lot of attention recently in computer vision because of its numerous applications in sports analytics, healthcare, autonomous systems, and surveillance (Sultani and Shah, 2021). To improve the performance and safety of the smart system and to recognize how people act, this stage is acknowledged as crucial. We demonstrate how this helps people make better decisions in difficult situations (Yadav et al., 2023). The adoption of HAR systems is increasing due to recent advancements in UAV technology, commonly known as drones. Equipped with high-quality cameras and precise flight controls, drones capture more flexible and dynamic data compared to ground-based cameras. Modern drones play a crucial role in various applications, including search and rescue operations, disaster response, military surveillance, traffic management, and crowd monitoring. Their ability to autonomously access remote areas makes UAV technology a valuable tool for efficient monitoring and data collection.

The implementation of HAR in drone systems introduces new challenges. Multiple viewing points, shifting settings, and shifting light conditions are all part of the video that drones capture (Perera et al., 2020; Ahmad et al., 2022). As subjects appear smaller and harder to distinguish from multiple camera angles, the combination of drone altitude and viewing angle poses challenges for action detection methods. Developing algorithms capable of simultaneously tracking multiple individuals performing tasks is essential. The integration of drones with HAR holds great potential for advancing smart environment applications, autonomous navigation systems, and public safety solutions.

Our methodology presents a framework that expands the precise recognition of multi-person actions in UAV videos. Our system combines robust preprocessing and multi-level feature extraction with classifiers to ensure accurate performance in diverse and dynamic aerial environments (Barekatain et al., 2017). The system begins with a preprocessing pipeline that enhances UAV footage quality by reducing noise, removing background elements, and isolating human subjects. It then extracts human silhouettes and generates skeletal representations of 33 keypoints using MediaPipe Pose estimation. These skeletal features effectively capture spatial dynamics and temporal action sequences.

Our approach leverages two types of feature extraction strategies: full-body features and keypoint-based features. The full-body features applied are Fourier Descriptors, Distance Transform, and AKAZE descriptors because they recognize body structures and movement patterns. Keypoint features capture meaningful data from the human body through discrete anatomical landmarks (keypoints). These features enhance the accuracy of human motion analysis by effectively tracking spatial and temporal keypoint relationships (Öfverstedt et al., 2020). The process combines both feature types to maximize system performance. Keypoint-based features, such as 0–180° intensity features, keypoint-based motion histograms, and multi-point autocorrelation are used. For classification, we employ three deep learning classifiers: Deep Belief Networks (DBN), Convolutional Neural Networks (CNN), and Recurrent Neural Networks (RNN) which utilize gradient descent as optimization. Deep learning classifiers have been specifically chosen to efficiently analyze spatial and temporal features, ensuring robust classification of multi-person actions in UAV-captured videos. The proposed framework demonstrates incredible potential for applications in intelligent systems, such as public safety monitoring, disaster management, and sports performance analysis, where drones play a vital role in capturing actions in complex aerial scenarios.

The key contributions of this work are as follows;

- Developed an adaptive deep learning-based framework for multi-person action recognition in UAV-captured videos, addressing issues such as distinct perspectives and changing backgrounds.
- Proposed a multi-level feature extraction approach, utilizing full-body features (Fourier Descriptors, Distance Transform, AKAZE) to capture movement patterns and keypoint-based features (0–180° intensity features, keypoint-based motion histograms, multi-point autocorrelation) to analyze spatial and temporal features.
- Implemented gradient descent optimization to fine-tune advanced deep learning classifiers—Deep Belief Networks (DBN), Convolutional Neural Networks (CNN), and Recurrent Neural Networks (RNN)—for accurate action classification.
- Evaluated the framework on two benchmark datasets MOD20 and Okutama-Action, achieving accuracies of 91.50 and 89.71%, respectively, demonstrating its effectiveness across aerial environments with diverse viewpoints and scenarios.

This approach demonstrated a major advancement in multi-person action recognition from UAV-captured videos, tackling the issues posed by dynamic aerial imagery, changing viewpoints, and environmental complexities.

The rest of the paper is structured as follows: Section 2 includes a comprehensive analysis of related work in multi-person action recognition and UAV-based applications. Section 3 explains the proposed methodology. Section 4 outlines the experimental setup, datasets, and evaluation measures employed, followed by a detailed analysis of the results in Section 5. Finally, Section 6 concludes the paper and suggests possibilities for future work in advanced feature integration and scalable frameworks for multi-person action recognition utilizing UAVs.

## 2 Literature review

The latest computer vision developments help stronger recognition of human actions from UAV images. Researchers split their studies into machine learning and deep learning methods. The two strategies worked together to make progress in the research area.

### 2.1 UAV imagery over machine learning

Machine learning-based approaches for human action recognition in UAV imagery rely on feature extraction and classification models rather that automatic feature learning. A machine learning method designed by Abbas and Jalal (2024) uses UAV videos to recognize human activities through multiple features extraction and classification operations. Uses UAV videos to recognize human activities through multiple feature extraction and classification operations. YOLOv5 first detects humans, followed by pose estimation, which extracts key points representing body joints. The system computes angular relationships, distance values, and 3D point cloud features using the extracted key points. The feature space is further enhanced through Linear Discriminant Analysis (LDA), which reduces dimensionality and improves feature separability. The system utilizes a multi-class Support Vector Machine as its final stage for action classification. The proposed method effectively identified human activities when tested on the Drone-Action dataset, demonstrating successful results. The work implements a conventional machine learning framework that combines manually extracted features alongside optimization steps instead of using automatic deep learning feature extraction along with optimization (Arunnehru et al., 2022). 2D and 3D DIDGP descriptors with spatiotemporal interest points to create a system for detecting human activity. Prior to providing advantages for human action recognition, the research methodology combines DCT and DWT transformations and employs PCA-based dimension reduction in feature extraction. When evaluating the UT-Interaction dataset using SVM and RF classifiers, the verification procedure achieved high accuracy, yielding greater precision than previously employed techniques. According to research findings, robust video-based human activity detection systems can be effectively solved using human-developed space–time properties.

### 2.2 UAV imagery over deep learning

Deep learning enhances UAVs’ ability to interpret activities by directly analyzing video data from aerial imagery, without relying on specific distinguishing features. CNNs, combined with transformer-based deep learning approaches, excel at detecting small moving objects, regardless of camera angle or occlusion. In drone surveillance applications, these methods outperform traditional machine learning techniques. Because of its excellent quality and low human component, research is increasingly concentrated on creating new architectural ways for UAV video analysis. Drone-HAT, a Hybrid Attention Transformer (HAT) framework for identifying multiple subjects’ behaviors from UAV surveillance video data (Khan et al., 2024). The study highlights the difficulties in tracking human movements in expansive drone photos that disperse over numerous small objects the size of humans. The system uses a Vision Transformer model for action detection, YOLOv8 for object identification, and DeepSORT for tracking. The study introduces a novel feature fusion technique that efficiently extracts highly accurate data while minimizing computational costs. In drone surveillance applications, transformer networks leverage multi-level attention mechanisms to precisely monitor and classify human behavior. The findings highlight how attention-based networks effectively handle the core video processing requirements for tracking multiple targets performing diverse actions (Gundu and Syed, 2023), a deep learning framework for UAV recording human activity identification is created by combining HOG, Mask-RCNN, and Bi-LSTM. Small moving objects in UAV surveillance footage move across complicated environments at varying speeds, making it challenging to identify human movements. The goal of the study is to resolve this specific problem. This approach works on images that detect both edges and shapes before HOG measurement. The Mask-RCNN model can identify individual subjects in drone video frames thanks to its remarkable feature map extraction results. In order to identify temporal–spatial activity patterns between frames that come before and after one another, the Bi-LSTM network examines video frames. The capacity of this method to identify various human activities on YouTube aerial footage is demonstrated by experience-based data. The study demonstrates how feature descriptors and deep learning enhance UAV systems’ capacity to identify human activity in the air. To develop a technique for video classification that efficiently extracts spatial–temporal data, the authors (Vrskova et al., 2023) combined 3DCNN with ConvLSTM. By using the well-known datasets LoDVP and UCF50, the researchers show the effectiveness of their approach. To fully illustrate the benefits of the combination of 3DCNN and ConvLSTM, the study needs further details on why it performs better than standard video categorization techniques. When the study broadened its data analysis methodology with authentic real-world datasets that go beyond recent survey results, the testing procedure would become more credible. Integrating 3DCNN and ConvLSTM enhances deep learning systems’ ability to classify videos more effectively. This approach leverages advanced neural network architectures to optimize performance in video analysis. To detect different people and recognize their movements during airborne security operations, (Geraldes et al., 2019) used deep learning techniques in their UAV-based situational awareness system, PAL. To detect many people and recognize their actions, the system uses deep learning models POINet and ActivityNet, which use LSTM. With the aid of the Pixel2GPS converter, the PAL system employs near real-time operations to convert UAV video feed frames into GPS locations in real time for individuals that have been detected. When our system was tested using the Okutama dataset, action identification performance held steady even when the UAV flight altitude and camera angle changed. The PAL system’s requirements are largely determined by the computational demands of deep learning models, making efficient hardware essential for optimal performance. The study demonstrates that deep learning is effective for UAV-based multi-person action recognition; nevertheless, more performance improvements and environmental modifications are required. MITFAS (Mutual Information-Based Temporal Feature Alignment and Sampling) was proposed by Xian et al. (2024) to recognize human actions in UAV recordings. This approach addresses three main issues: obscured items, drone shifts, and viewpoint alterations, as well as the effects of drone movement on backdrop elements. The method locks synchrony between temporal domain variables that contain action information by using mutual knowledge; as a result, recognition models only consider human motions. The suggested approach determines which UAV video frames provide the greatest advantages for video stream analysis by using joint mutual information. The suggested approach for evaluation in various UAV action recognition datasets is included into the deep learning model. Aerial Polarized-Transformer Network (AP-TransNet), created by Dhiman et al. (2024) used aerial video cameras to identify human movements. This system handles occlusion problems, complicated backgrounds, and various view angles by combining spatial and temporal information. By managing relevant or irrelevant information and efficiently filtering details, the Polarized Encoding Block (PEB) is the primary feature representation augmentation tool that improves action recognition performance. Inception pre-trained modules help in the framework’s ability to recognize spatial patterns, while transformer-based modeling allows it to comprehend temporal patterns across video shots. Functional tests and extensive trials have shown the system’s resilience, demonstrating its effectiveness and capability for drone-based HAR applications, especially surveillance and monitoring systems. In order to identify “who is doing what?” (Yang et al., 2019) proposed a new algorithm that can identify several atomic visual actions in aerial security footage. In order to process high-resolution images and generate effective detection recommendations, a Clustering Region Proposal Network (C-RPN) functions inside an integrated framework.

The system also integrates action recognition, multi-object tracking, and object detection. Prior to a 3D ConvNet classification step, the Spatio-Temporal Attention Module (STAM) directs the target individuals into spatiotemporal tubes using its focus mechanism. The suggested framework demonstrated exceptional performance for simultaneous action recognition, tiny object handling, and drone movement on the Okutama-Action dataset.

## 3 Materials and methods

### 3.1 System methodology

**Figure 1:**
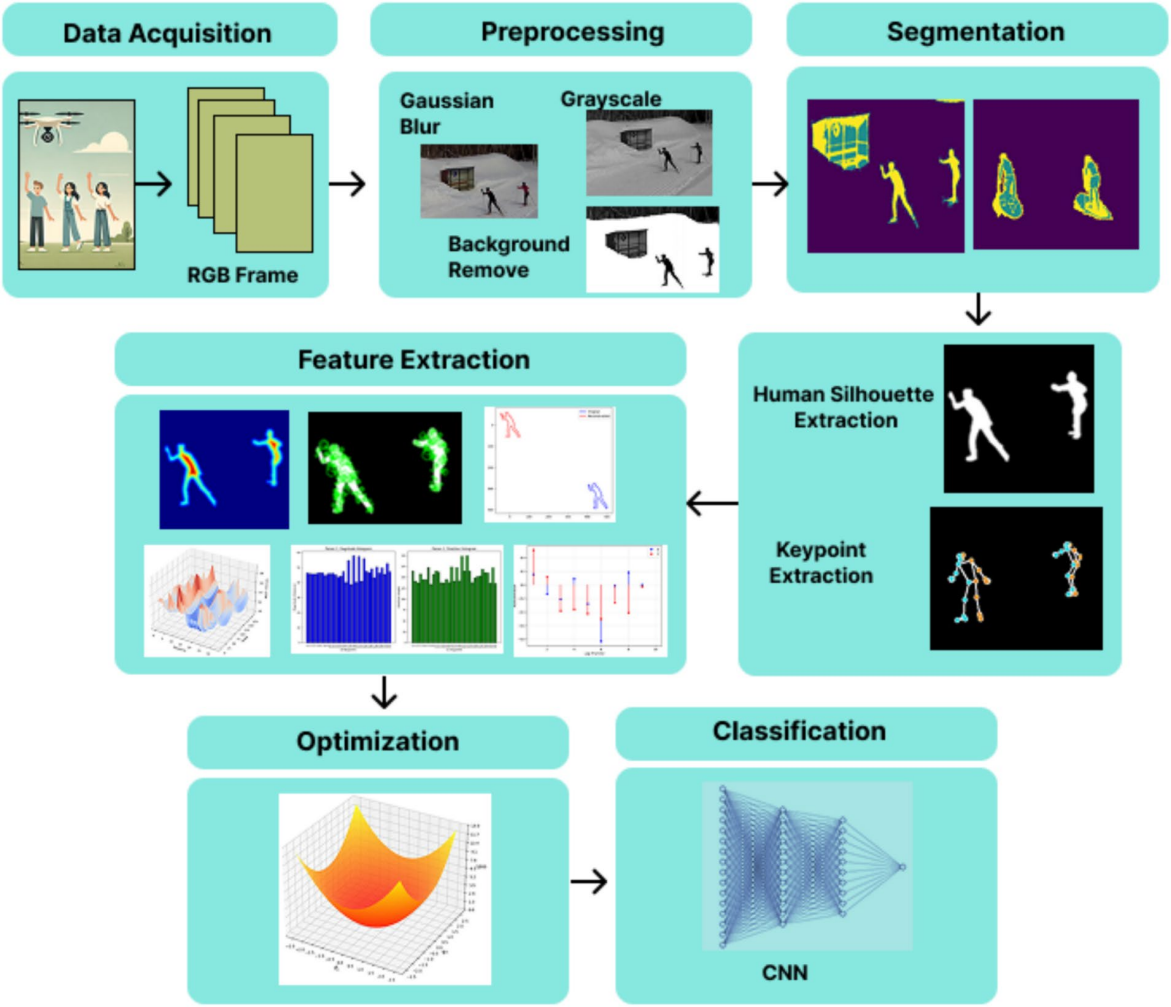
Detailed architecture of the proposed system for multi-person action recognition.

The proposed UAV-captured video multi-person action recognition in the UAV-captured videos adopts a structural approach designed to address the unique challenges of aerial imagery. The methodology emphasizes both feature extraction strategies to maximize system accuracy. The pipeline begins with preprocessing procedures, including noise reduction through Gaussian blur and grayscale conversion and background removal operation. This is followed by segmentation using the Gaussian Mixture Model (GMM) to obtain human silhouettes. Subsequently, a skeletal model is constructed to represent the keypoints. Feature extraction is achieved through two methods: full-body features, which capture overall movement patterns, and keypoint-based features, which emphasize motion dynamics using landmarks within the body. A gradient descent optimizer is used for efficient optimization, followed by classification utilizing three deep learning classifiers: DBN, CNN, and RNN. This pipeline allows exact multi-person action recognition across diverse UAV scenarios. Figure 1 illustrates the proposed system architecture.

### 3.2 Pre-processing

The preprocessing stage prepares UAV-captured video frames which enables robust and accurate multi-person action recognition. During this processing stage, the system addresses essential challenges related to noise, dynamic backgrounds, and lighting. The preprocessing steps are performed sequentially as follows: Frame extraction, Gaussian blur, Grayscale conversion, and background removal. In the first step frame extraction, video data is divided into distinct frames to process each image separately. This stage turns continuous video streams into discrete image sequences, giving a structured input format for future preprocessing and feature extraction. The retrieved frames constitute the foundation for further analysis. Next, Gaussian blur is applied to reduce image noise and smoothen the frame for better feature extraction. Gaussian blur acts by avenging the pixel brightness in the neighborhood of each pixel using a Gaussian kernel. The blurred intensity value $F_{i}^{\prime }\left(x,y\right)$ at pixel location *(x, y)* is calculated as Equation 1;


(1) $$F_{i}^{\prime }\left(x,y\right)=\frac{1}{2\pi \sigma^{2}}\overset{k}{\underset{u=-k}{\sum }}\overset{k}{\underset{v=-k}{\sum }}F_{i}\left(x+u,y+v\right)\;.\;e^{-\frac{x^{2}+y^{2}}{2\sigma^{2}}}$$

Here, σ represents the standard deviation of the Gaussian function, controlling the amount of smoothing, and *k* determines the kernel size. This process eliminates high-frequency noise while maintaining edge details, which is essential for further segmentation and feature extraction.

Following noise reduction, the frames are converted to grayscale. The frames are transformed from RGB to grayscale, simplifying the data by minimizing the color channels while preserving the intensity information. This phase decreases computational complexity and guarantees the preservation of brightness changes, which is essential for identifying human actions. The grayscale intensity *G_i_(x, y)* at a pixel position *(x, y)* in the frame $F_{i}^{\prime }$ is computed as Equation 2;


(2) $$G_{i}\;\left(x,y\right)=0.2989\;.\;R\left(x,y\right)+0.5870\;.\;G\left(x,y\right)+0.1140\;.\;B\left(x,y\right)$$

Where, $R\left(x,y\right)$ , $G\left(x,y\right)$ , and $B\left(x,y\right)$ represent the red, green and blue color channels of the pixel, respectively. This step reduces the computational complexity by retaining the luminance information while discarding color data.

**Figure 2:**
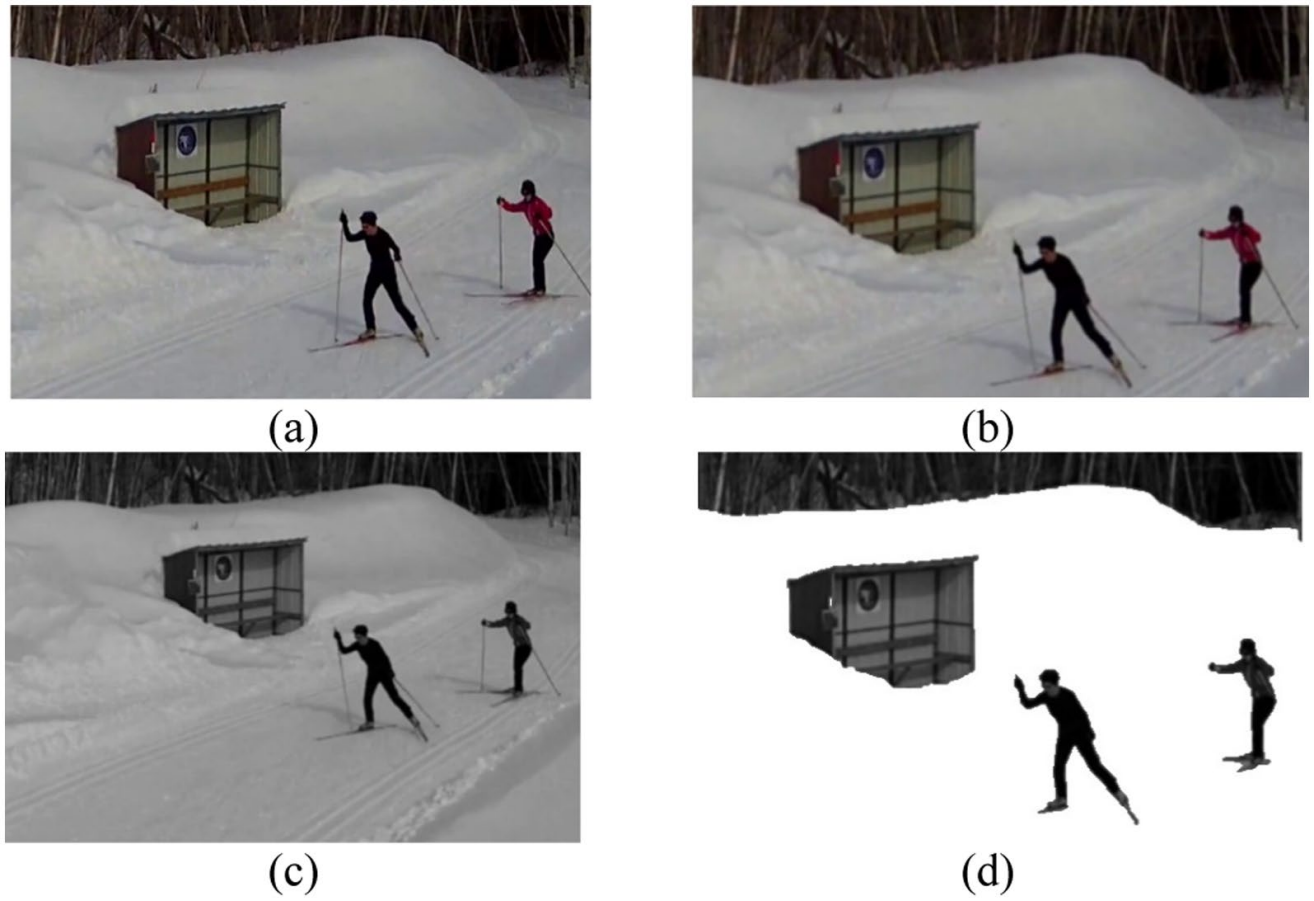
Sequential depiction of preprocessing stages: **(a)** Original frame, **(b)** Gaussian Blur, **(c)** Grayscale, and **(d)** Background removed.

The final steps achieve maximum noise reduction to enhance human visibility within UAV video recordings. The goal during this step is to eliminate dynamic and complex background components including vehicles that move along with vegetation and shadows that cause noticeable noise. The isolation process for human shapes combined with detail removal focuses the observation exclusively on human motion. Subject isolation becomes essential when there are multiple humans since it helps achieve accurate segmentation and feature extraction in later processing steps. The results of these preprocessing steps, highlighting the progressive refinement of the input frames, are illustrated in Figure 2.

### 3.3 Gaussian Mixture Model segmentation

The segmentation process employs Gaussian Mixture Model (GMM) analysis to partition images, to distinguish human subjects from background (Xing et al., 2019; Hou et al., 2023). The isolation of human subjects from dynamic environments with automobiles, plants, and shadows is necessary for UAV image-based human activity recognition. Image grayscale data transforms into a single-dimensional array format as the segmentation process starts through treating each pixel like a distinct data value. The analysis procedure applies Gaussian distribution mixture models to pixel intensity values. The GMM assumes that the pixel intensities are generated from a mixture of *K* Gaussian components, and the probability density function for each pixel *I* is represented as Equation 3;


(3) $$p\left(x_{i}\right)=\overset{K}{\underset{k=1}{\sum }}\pi_{k}N\left(x_{i}\;|\;\mu_{k},\sigma_{k}^{2}\right)$$

Where $p\left(x_{i}\right)$ is the probability density function for pixel *i*, $\pi_{k}$ is the weight of the *k*-th Gaussian component, $N\left(x_{i}\;|\;\mu_{k},\sigma_{k}^{2}\right)$ is the Gaussian distribution with mean $\mu_{k}$ and variance $\sigma_{k}^{2}$ and *K* represents the number of Gaussian components. The Expectation–Maximization (EM) algorithm is employed to estimate the parameters $\mu_{k},\sigma_{k}^{2}$ , and $\pi_{k}$ of the Gaussian components. In the E-step, the algorithm iterates between two main steps: the E-step and M-step. In the E-step, the algorithm calculated the posterior probability $\gamma_{ik}$ , which indicates the probability that pixel *i* belongs to the *k*-th Gaussian component. This probability is computed using Equation 4;


(4) $$\gamma_{ik}=\frac{\pi_{k}N\left(x_{i}\;|\;\mu_{k},\sigma_{k}^{2}\right)}{\overset{K}{\underset{j=1}{\sum }}\pi_{j}N\left(x_{i}\;|\;\mu_{j},\sigma_{j}^{2}\right)}$$

The model runs these sequential steps through multiple iterations until parameters reach convergence at which point no substantial changes emerge. When algorithm convergence occurs, the model selects the most probable Gaussian distribution for assigning each pixel. A prediction exists for every pixel *i* through the model is given in Equation 5;


(5) $${\hat{z}}_{i}=\arg\max_{k}\gamma_{ik}$$

Where ${\hat{z}}_{i}$ represents the predicted label for pixel *i*, and each pixel is assigned to the Gaussian component with the highest responsibility.

The GMM segmentation method produces as its final result an image with pixel data that receives labels connected to individual Gaussian components. Human figure detection becomes possible through subsequent analysis of these segmented regions. GMM segmentation identifies regions of human figures through analysis of pixel intensity statistics and distance-level characteristics. The segmentation approach contributes significant value toward UAV-based human detection operations that face dynamic changes in background characteristics. Through its statistical distribution approach which allows pixel values to be represented by Gaussian distributions GMM achieves robustness against variations in the environment thus enabling successful human subject segmentation.

**Figure 3:**
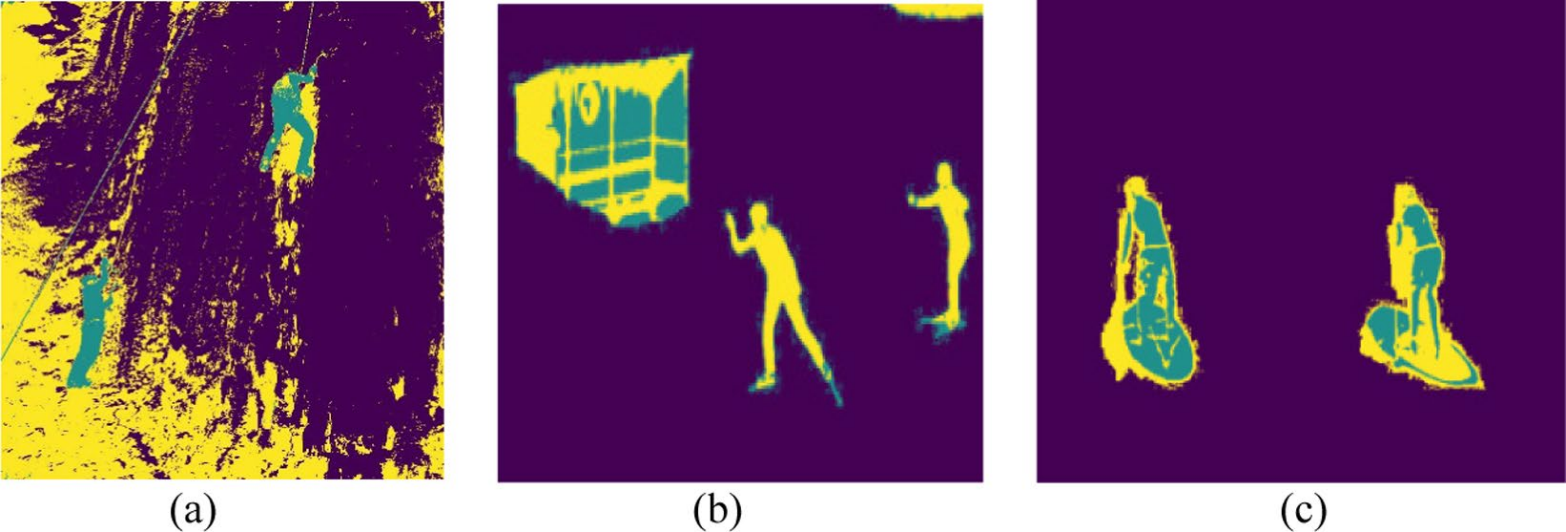
GMM segmentation results of three different action classes: **(a)** Rock Climbing, **(b)** Skiing, and **(c)** Standup Paddling.

This GMM-based segmentation approach plays a key role in background noise reduction as well as human subject enhancement to optimize image processing throughout the human action recognition pipeline. The method facilitates optimal segmentation thus enabling precise extraction of human silhouettes and significant image features. Figure 3 shows the results of GMM segmentation for three different action classes.

### 3.4 Human silhouette extraction

The human silhouette extraction follows Gaussian Mixture Model (GMM) segmentation for improved human figure segmentation while removing background noises. The human shapes require complete separation at this point to achieve precise feature extraction in the following process (Prakash et al., 2018; Pervaiz et al., 2021). The output segments from GMM construct regions whose pixels indicate various brightness values for each categorized area. Human silhouette retrieval requires first conducting thresholding on the segmented image to identify human regions from other image parts. This binary mask is represented in Equation 6:


(6) $$B\left(x,y\right)=\{\begin{array}{c} 1,\mathrm{if}\;S\left(x,y\right)=C_{h}\\ 0,\mathrm{otherwise} \end{array}$$

**Figure 4:**
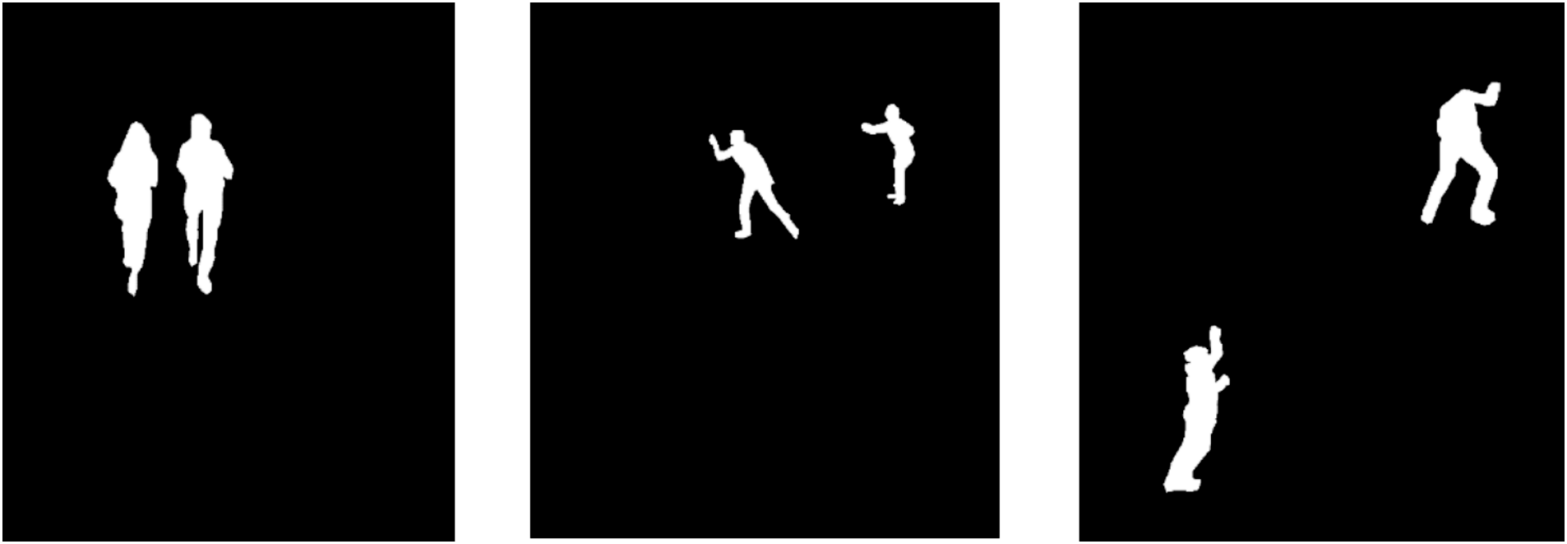
Results of human silhouette extraction, showcasing refined human shapes for three distinct action classes.

Where *S (x, y)* represents the segmented image, and $C_{h}$ denotes the intensity corresponding to the human class. To remove minor artifacts and increase silhouette boundary clarity morphological processes called erosion and dilation work next to thresholding. These methods clean the binary mask by reducing noise and filling minor gaps to obtain a refined silhouette. The system selects the largest connected connective shape because it matches the human body figure. The solution separates the silhouette through a process that discards disconnected regions and small background elements. Following connected component identification, the largest visual area becomes the human silhouette. The generated silhouette presents a clean, isolated binary format of the human shape, which facilitates the system’s focus on human movements independent of background distractions. This initial outline maintains its significance throughout the entire action recognition process because it produces reliable human figure features. Figure 4 shows the results of human silhouette extraction for three different action classes.

### 3.5 Keypoint extraction

This step involves identifying and extracting key body landmarks from 2D image data using skeletonization techniques. A skeletal representation is generated by detecting key points and connecting body joints, defining posture and spatial orientation (Doan, 2022). Through the MediaPipe Pose library, a pre-trained model detects the human body’s 33 landmarks to generate extraction results (Ma and Tran-Nguyen, 2024). The pose estimation algorithm detects landmarks while providing then as normalized 2D coordinates *(x, y)* along with visibility *(v)* to determine the landmarks detection accuracy. These normalized coordinates are calculated as in Equations 7, 8:


(7) $$x_{\mathrm{norm}}=\frac{x_{\mathrm{pixel}}}{\mathrm{width}\;}$$

(8) $$y_{\mathrm{norm}}=\frac{y_{\mathrm{pixel}}}{\mathrm{height}\;}$$

Where $x_{\mathrm{pixel}}$ and $y_{\mathrm{pixel}}$ represent the pixel locations of the landmark in the original image, and width and height are the dimensions of the image. The landmarks detected are represented as in Equation 9:


(9) $$L=\left\{\left(x_{i},y_{i},v_{i}\right)\;|i=1,2,3,\ldots \ldots N\right\}$$

**Figure 5:**
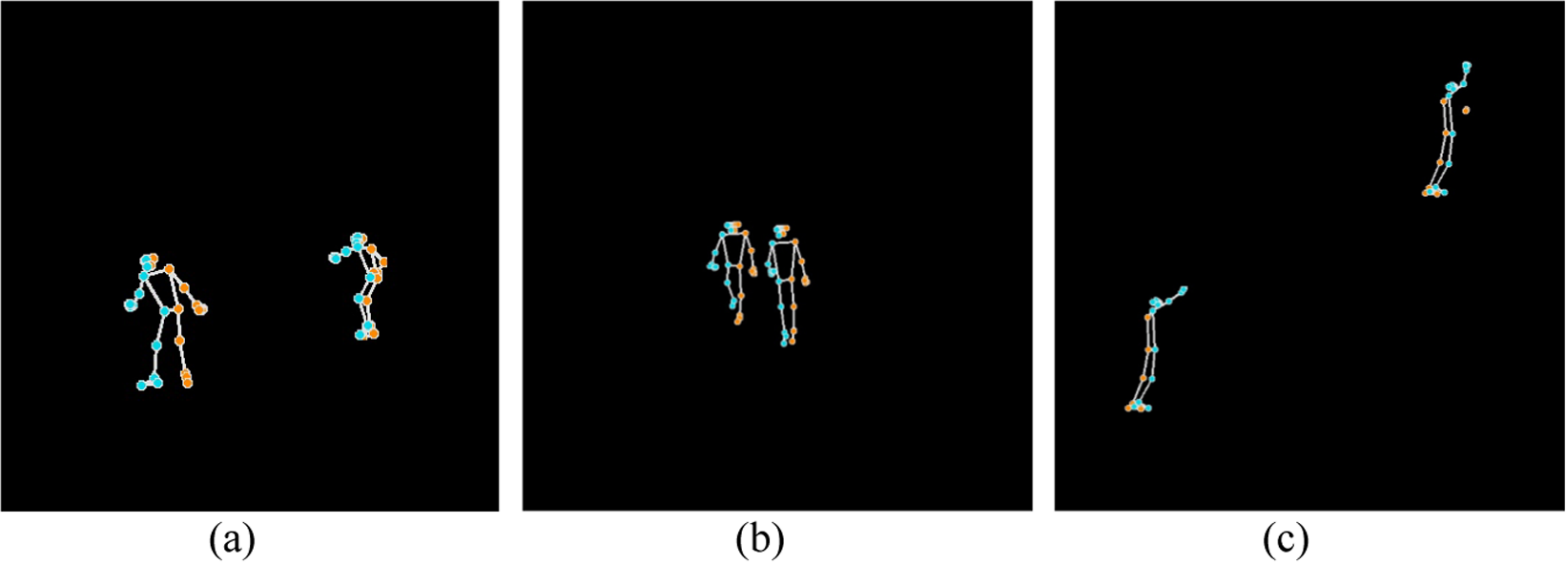
Skeletonized representation of three different action classes: **(a)** Skiing, **(b)** Backpacking, and **(c)** Rock climbing.

Where *N* is the total numbers of landmarks 33, $x_{i}$ and $y_{i}$ are the normalized coordinates of the *i*th landmark, $v_{i}$ is the visibility score. Once the landmarks are detected, they are connected based on anatomical relations to form the human skeleton. The skeleton is mathematically modeled as graph *G(V, E)*, where *V* is the set of landmarks, and *E* is the set of edges connecting the landmarks, as defined by anatomical connections. The skeletal structure develops through relationships between body points like shoulder-elbow joints or hip-knee joints and these interactions become visible through connecting landmarks with lines. This skeletal representation maintains its significance for future feature extraction and action classification work because it preserves normalized 2D landmark coordinates. The skeletal representation offers an efficient computational method to model human body spatial arrangements. Figure 5 shows the skeletal representation of three different action classes.

### 3.6 Feature extraction

Feature extraction is the primary operational stage of the proposed multi-person action recognition system. The technique next converts the preprocessed data into useful feature representations for categorization. The recognition system uses both full-body features to detect the entire body’s movement and keypoint-based features to track specific body spots. With its full body and keypoint-based feature interpretation of human movements, the identification system achieves strong results for complicated UAV situations.

#### 3.6.1 Full-body features

The proposed multi-person action recognition system uses full-body measurements to identify human movement and shape characteristics. By continuously monitoring key movement characteristics, these monitoring features allow for a comprehensive understanding of human body dynamics over the global dimension of UAV operations. In order to distinguish between action-relevant motion alterations that result in accurate identification, the system assesses the shapes of the human body. Three full-body features are used by the system: AKAZE, Distance Transform, and Fourier Descriptor. The system can recognize different behaviors in complex aerial environment settings because of these properties, which also allows it to execute robust representation processing.

##### 3.6.1.1 AKAZE feature

AKAZE (Accelerated-KAZE) extracts robust keypoints quickly through its detection and description capabilities. Because the Perona-Malik anisotropic diffusion preserves relevant image features in addition to noise reduction (Tareen and Saleem, 2018; Hu et al., 2020), AKAZE is able to create a nonlinear scale-space. The diffusion sequence happens following Equation 10:


(10) $$\frac{\partial L}{\partial t}=div\left(c\left(x,y,t\right)\nabla L\right)$$

While the image gradient *∇L* serves as a crucial component of the calculation, the image *L(x, y, t)* at various scales interacts with the conductivity function c(x, y, t). Because of its effective boundary preservation, AKAZE is able to keep edge details better than other descriptors, particularly in human silhouette analysis, when nonlinear diffusion is used instead of Gaussian blurring. In order to determine its position, AKAZE keypoint detection looks for regions with significant contrast and texture fluctuations using the Hessian matrix determinant value. Equation 11 uses second-order derivatives to calculate the determinant:


(11) $$\det\;\left(H\right)=L_{xx}L_{yy}-{\left(L_{xy}\right)}^{2}$$

**Figure 6:**
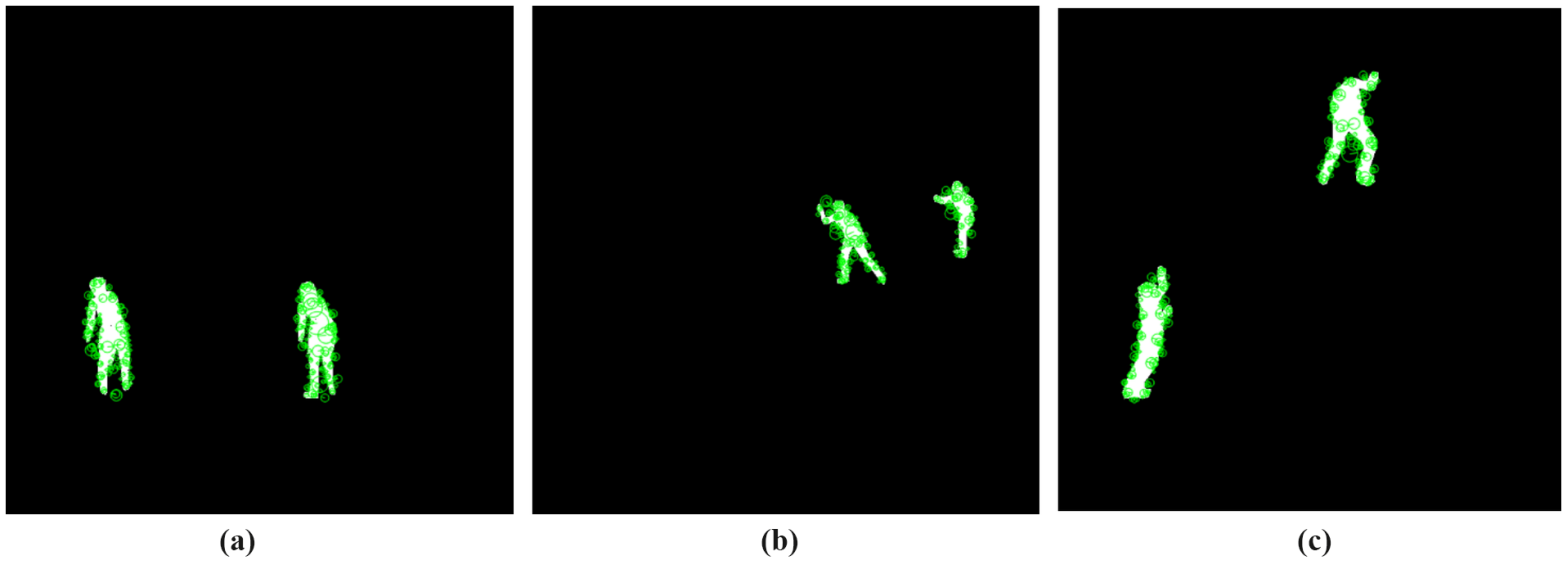
AKAZE feature detection on human silhouettes for three different action classes: **(a)** Standup Paddling, **(b)** Skiing, and **(c)** Rock climbing.

Where $L_{xx}$ and $L_{yy}$ represent the second-order derivatives along the *x-* and *y-*axis, while $L_{xy}$ represents the mixed derivative. The detection of keypoints depends on identifying local maximum values from the determinant function which operates across various scale levels for maintaining robust detection through transformation like scaling and rotation. AKAZE detects keypoints before employing its Modified Local Difference Binary (MLDB) method to generate descriptors through keypoint neighborhood intensity comparison analysis. The descriptors serve as matches during the comparison of images. When AKAZE analyzes human silhouettes, it generates effective shape recognition and structural information which produces dependable features for subsequent analysis of action recognition. Figure 6 shows AKAZE feature detection on human silhouettes for three action classes.

##### 3.6.1.2 Distance transform feature

The Distance Transform is a mathematical algorithm that calculates the pixel-to-boundary distance for each point in binary images, determining the shortest possible edge length. It effectively detects spatial and geometric patterns in human silhouettes (Lindblad et al., 2020), making it valuable for recognizing body structures and motion dynamics in action recognition systems (Navarro et al., 2019; Tayyab et al., 2025). The distance transform operates on the human silhouette *S(x, y)* which contains white human shapes with values *S(x, y) = 255* and black background pixels with value *S(x, y) = 0*. It calculates pixel distances from human silhouette edges. Each pixel obtains its distance value by applying the Euclidean metric as shown in Equation 12:


(12) $$D\left(x,y\right)=\min_{\left(u,v\right)\in \mathrm{Boundary}\left(S\right)}\sqrt{{\left(x-u\right)}^{2}+{\left(y-v\right)}^{2}}$$

Where *(u, v)* represents the coordinates of the human silhouette boundary. The distance values undergo normalization to *[0, 255]* for feature extraction as follows as in Equation 13:


(13) $$D^{\prime }\left(x,y\right)=255.\frac{D\left(x,y\right)-D_{\text{min}}}{D_{\text{max}}-D_{\text{min}}}$$

**Figure 7:**
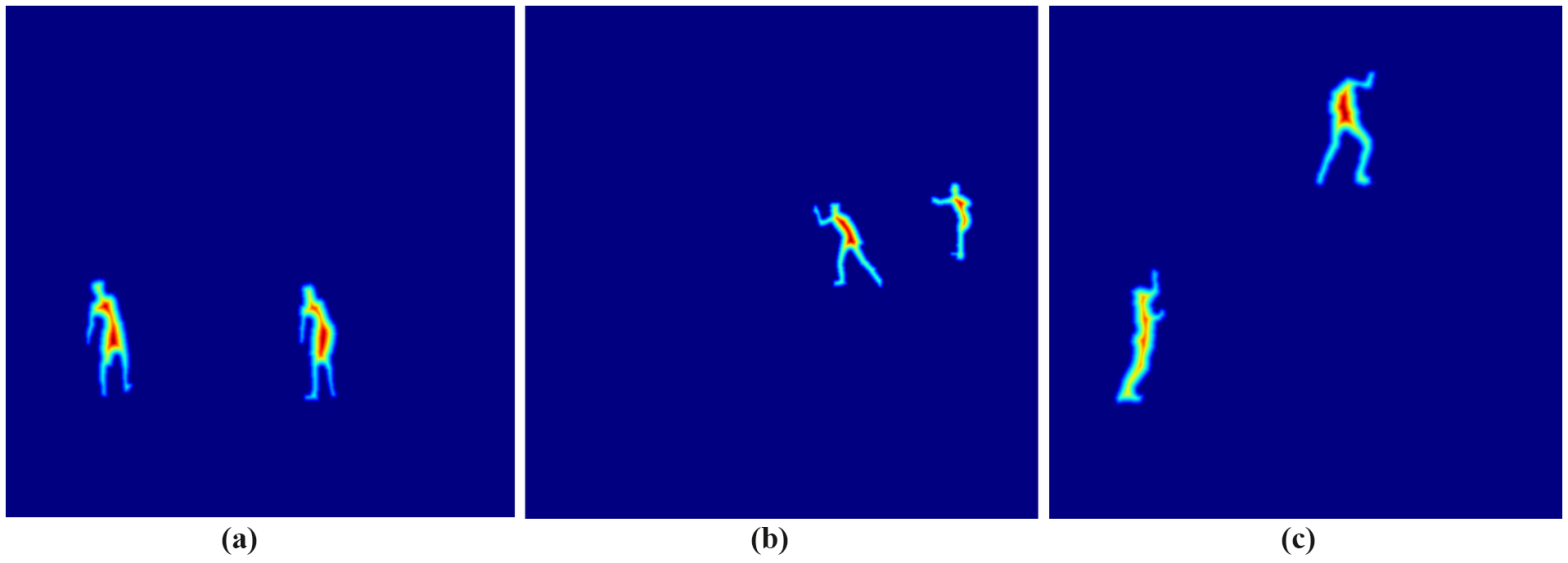
Distance transform feature extraction representation for three different action classes: **(a)** Standup Paddling, **(b)** Skiing, and **(c)** Rock climbing.

Where $D_{\text{min}}$ and $D_{\text{max}}$ are the minimum and maximum distance values within the computed distance map. The normalization technique creates uniform scaling of all distance values that maintain relative distances for suitable processing in subsequent stages. The results of the distance transform feature extraction for three different action classes are presented in Figure 7.

##### 3.6.1.3 Fourier descriptor

The transformation of object boundary data into frequency domain through Fourier descriptors (FDs) provides an effective method to represent object shapes. Shape analysis uses this method extensively to analyze human silhouettes because it shows stability across translation scaling and rotational changes. Fourier descriptors’ primary purpose includes converting shape boundary data into complex number sequences following the application of discrete Fourier transform (DFT) to reveal meaningful shape features (Yan et al., 2023; Saikia et al., 2021). Processing begins by extracting the boundary of the human silhouette. A representation of the boundary exists as a group of ordered contour points $\left(x_{n},y_{n}\right)$ that are placed in a 2D space. A sequence of complex numbers results from the conversion process of these points as shown in Equation 14:


(14) $$z_{n}=x_{n}+iy_{n},n=0,1,2,.\ldots \ldots \ldots .,N-1$$

Where *i* represents the imaginary unit. To extend FD into a 3D mathematical framework for MOD20 and Okutama-Action datasets, depth information (*z*) is normalized relative to the overall scale and incorporated into the contour representation as in Equation 15:


(15) $$\tilde{z}=z_{n}+\frac{z}{\max\left(z\right)}$$

Applying the Fourier transform to this sequence yields a set of Fourier coefficients as shown in Equation 16:


(16) $$Z_{k}=\overset{N-1}{\underset{n=0}{\sum }}z_{n}e^{-i2\pi kn/N},k=0,1,2,3,\ldots \ldots \ldots .,N-1$$

**Figure 8:**
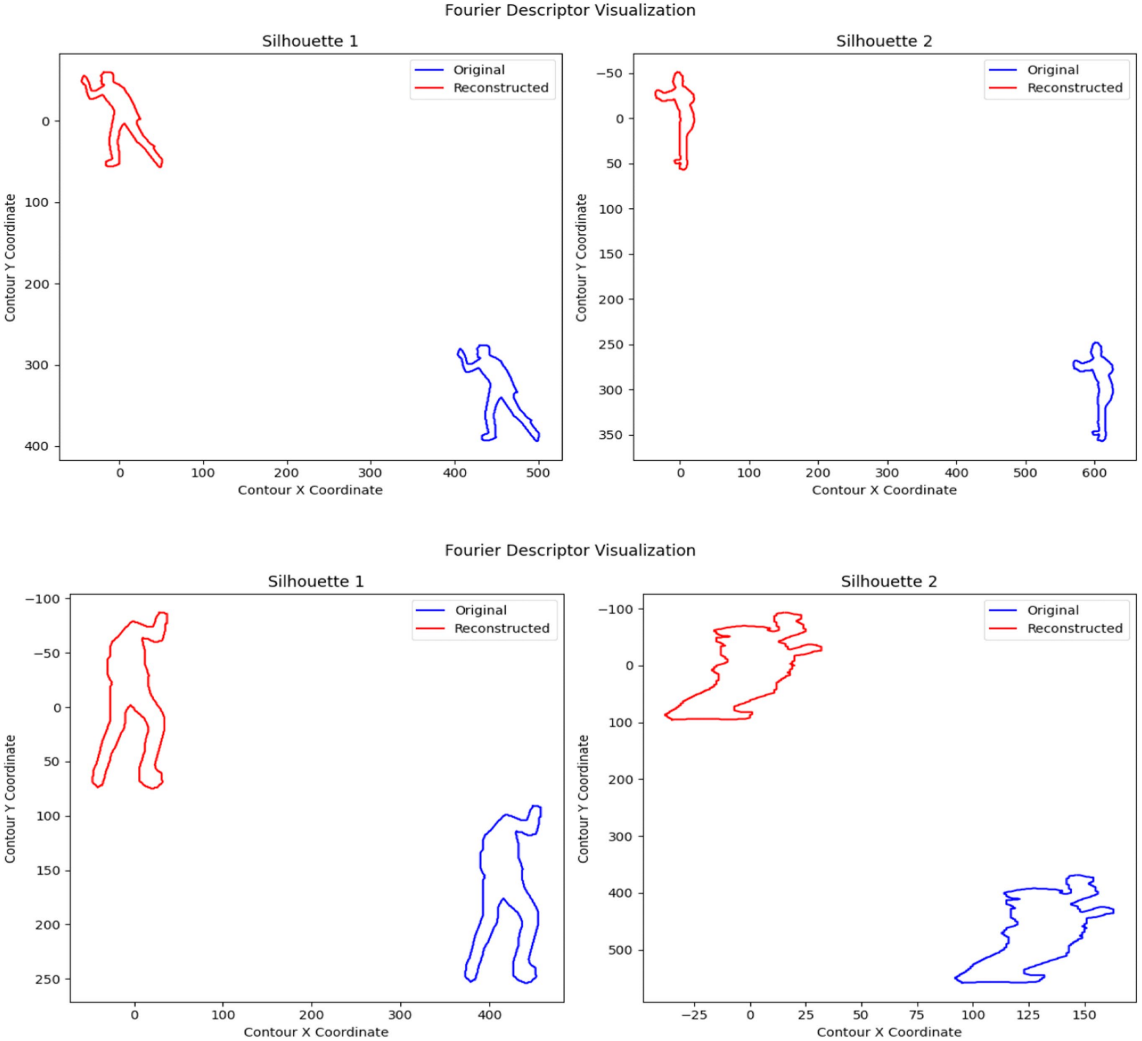
Fourier descriptor representation of human silhouettes of two different action classes: **(a)** Skiing, and **(b)** Rock climbing.

These coefficients represent the frequency components of the shape, capturing both global and local contour characteristics. The first Fourier coefficient (𝑍_0_) becomes zero to achieve translation invariance along with scale invariance achieved through coefficient normalization relative to the first non-zero coefficient and rotation invariance through phase alignment of Fourier coefficients. The transformation-invariant representation of objects comes from Fourier Descriptors (FDs). Translation invariance occurs when setting the initial Fourier coefficient value to zero and scale invariance results from normalizing coefficients relative to the first non-empty value. Human silhouette size variations do not affect the robustness of FDs. The contour reconstruction through Inverse Fourier Transform keeps low-frequency components to smooth noise yet maintain crucial shape information. FD operates on extracted 3D point clouds from UAV image silhouettes that come from both MOD20 and Okutama-Action datasets. FD provides stable and robust human action analysis by maintaining perspective invariant and rotation and scaling resistant 3D shape data representations. Silhouette images are processed to obtain contour data which permits FD computation and visualizes shape results during the implementation process. The technique proves efficient at detecting human body postures because of its capability to recognize actions. Figure 8 illustrates the Fourier descriptor-based shape representation for human silhouettes of two distinct human actions.

#### 3.6.2 Keypoint-based features

Keypoint-based features extract body movements and structural characteristics from human skeletal representations. The features derive from keypoint movements and their relative positions throughout a period of time to represent human movement’s characteristics. The proposed system implements multiple keypoint-based elements, including a 0–180° Intensity Feature, a Keypoint-Based Motion Histogram, and Multi-Point Autocorrelation Features. These features enhance the system’s ability to identify human motion patterns while emphasizing temporal and spatial information within human body movements.

##### 3.6.2.1 0–180° intensity feature

As a keypoint feature based on the skeletal model, the 0–180° Intensity Feature examines the pattern of angular intensity distribution in the skeletal model. The technique uses a Radon transform to calculate the mean intensity across angles ranging from 0° to 180°, facilitating providing structural patterns and directional information for human action (Akhter et al., 2021; Dwivedi et al., 2022). For a given patch centered at a keypoint, the Radon transform projects the intensity *f(x, y)* at an angle *θ* as shown in Equation 17:


(17) $$R\left(\rho ,\theta \right)=\int_{-\infty }^{\infty }\int_{-\infty }^{\infty }f\left(x,y\right)\delta \left(x\cos\theta +y\sin\theta -\rho \right)\mathrm{dxdy}$$

Where $R\left(\rho ,\theta \right)$ is the projection, ρ is the radial distance, and δ is the Dirac delta function ensuring projection alignment. To simplify interpretation, the mean intensity for each angle θ across all pixels in the patch is computed using Equation 18:


(18) $$M\left(\theta \right)=\frac{1}{N}\overset{N}{\underset{i=1}{\sum }}R\left(\rho_{i},\theta \right)$$

**Figure 9:**
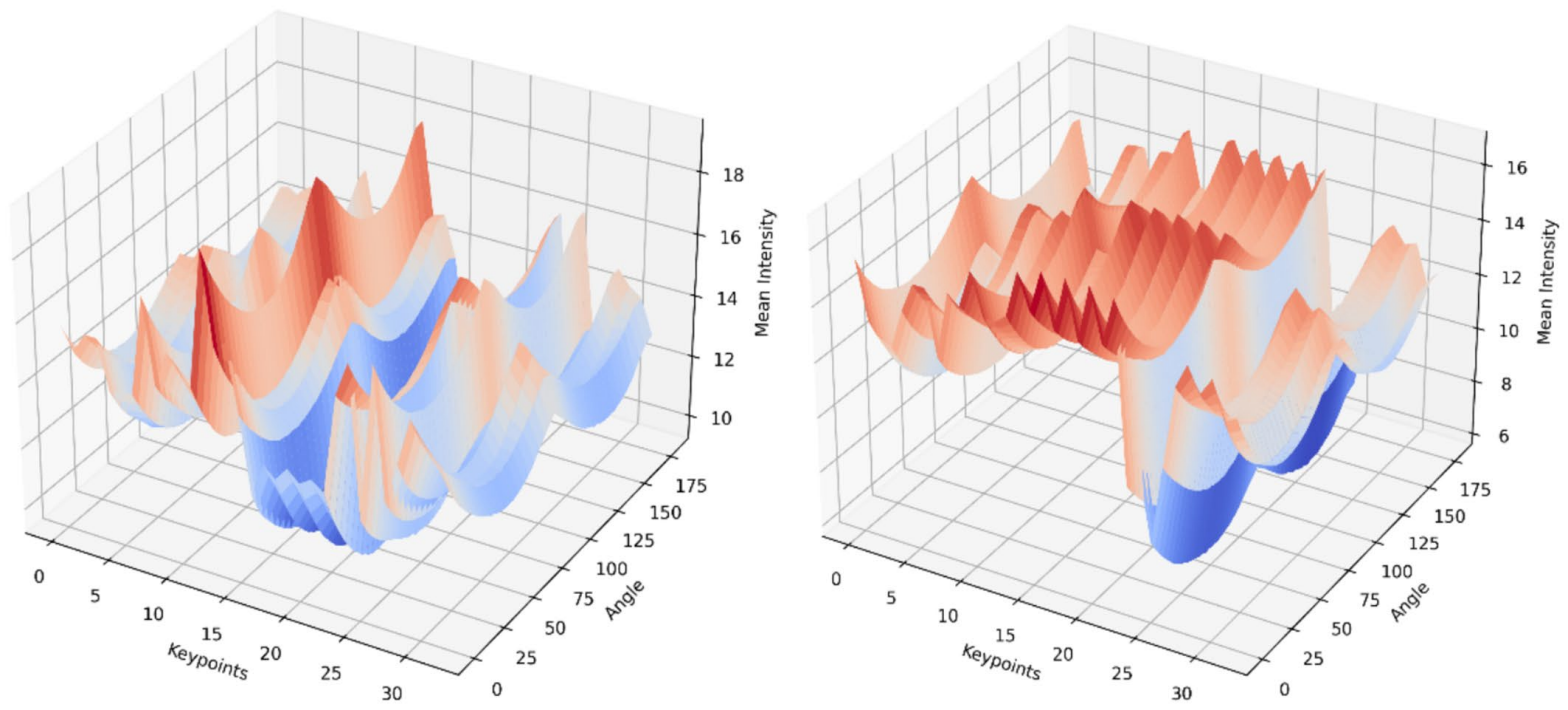
3D plot illustration of 0–180° intensity feature of two persons performing action.

Where N is the number pixels in the patch, and $R\left(\rho_{i},\theta \right)$ represents the Radon transform at $\rho_{i}$ . The analysis procedure progressively repeats for every skeletal component leading to the generation of intensity measurements across a 0–180° angle range. The profiles get assembled into 3D data that organizes its data points across keypoint positions with intensity measurement scales. Figure 9 shows 3D plot of the 0–180° intensity feature of two persons performing action.

##### 3.6.2.2 Keypoint-based motion histogram

The Keypoint-Based Motion Histogram feature processes sequential keypoint data from human skeletal data to extract movement data. A simple yet discriminative motion pattern representation is achieved by the feature by constructing direction and magnitude histograms from keypoint position change data from frames (Wahid et al., 2024; Bisht et al., 2024). The first step is extracting keypoints from consecutive frames. Each keypoint is represented by its spatial coordinates $\left(x_{i},y_{i}\right)$ . Motion vectors are computed to capture the displacement of each keypoint between two consecutive frames as shown in Equation 19:


(19) $$v_{i,t}=k_{i,t+1}-k_{i,t}=\left(x_{i,t+1}-x_{i,t},y_{i,t+1}-y_{i,t}\right)$$

Where $k_{i,t}$ represents the coordinates of keypoint *i* in the frame *t*. The magnitude of each motion vector quantifies the distance traveled by a keypoint as shown in Equation 20:


(20) $$M_{i,t}=\left\|v_{i,t}\right\|=\sqrt{{\left(x_{i,t+1}-x_{i,t}\right)}^{2}+{\left(y_{i,t+1}-y_{i,t}\right)}^{2}}$$

The direction of motion is determined as the angle of the motion vector as shown in Equation 21:


(21) $$\theta_{i,t}=\tan^{-1}\frac{y_{i,t+1}-y_{i,t}}{x_{i,t+1}-x_{i,t}}$$

**Figure 10:**
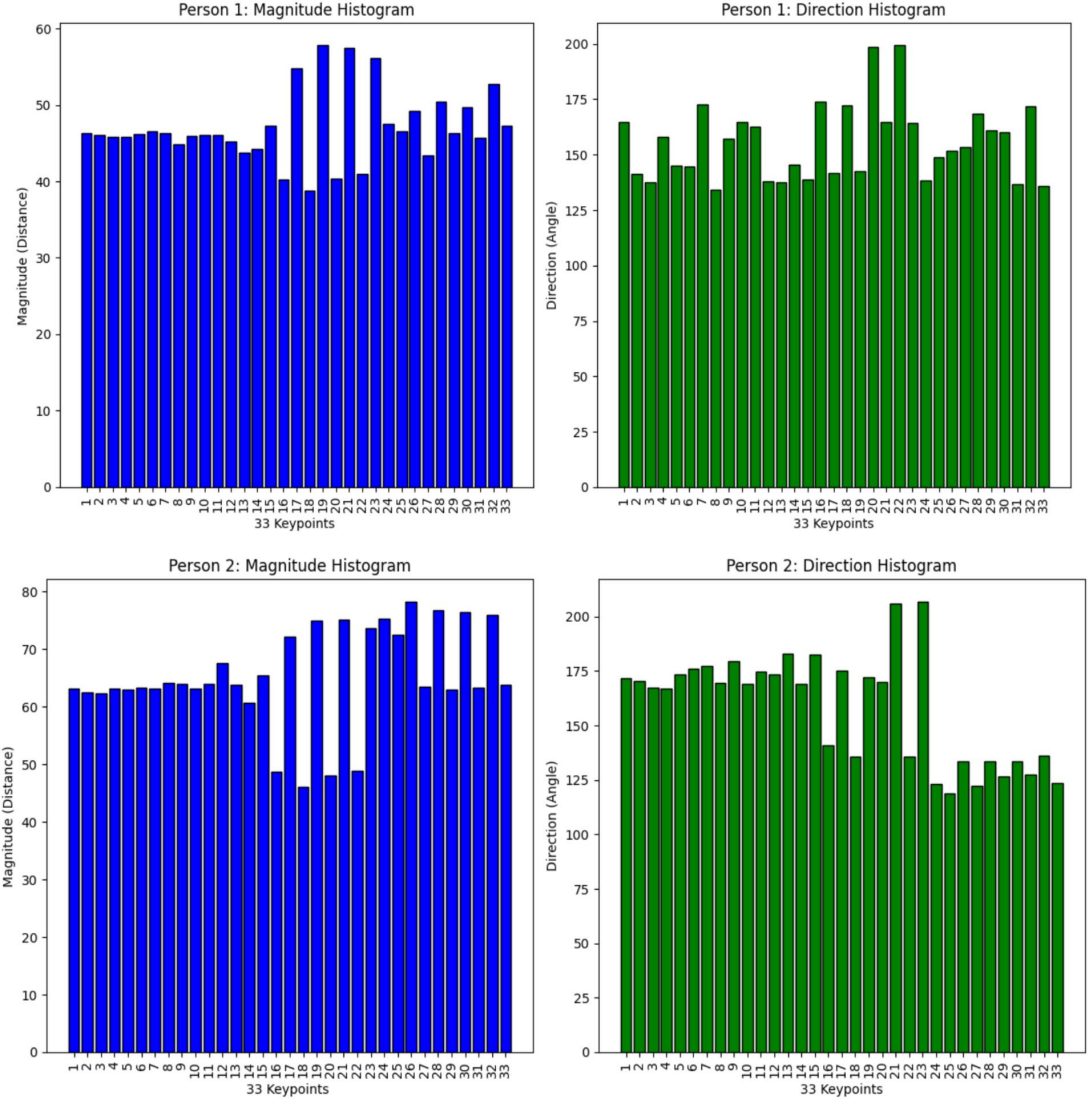
Magnitude and direction histogram of motion for 33 keypoints.

The analysis combines directional and magnitude data points from all frames and keypoints to generate histograms. The magnitude histogram emerges from dividing all magnitude values into defined bins whereas direction angles fall within [0°, 360°] angular bins to produce direction histograms. This procedure results in an efficient depiction of motion dynamics. Figure 10 shows the histogram of magnitude and direction of motion for 33 keypoints.

##### 3.6.2.3 Multi-points autocorrelation features

We utilized the Multi-Points Autocorrelation Function to analyze temporal patterns in human movement. This technique quantifies the self-similarity of keypoint movements over time intervals, identifying repetitive human actions (Gochoo et al., 2021; Ma and Zheng, 2024). The keypoint time-series autocorrelation measure determines the relationship between two points in a dataset based on a specified time lag. The autocorrelation for a time series signal *x(t)* of *N* points appears in the Equation 22:


(22) $$ACF\left(l\right)=\frac{\overset{N-l}{\underset{t=1}{\sum }}\left(x\left(t\right)-\bar{x}\right)\left(x\left(t+l\right)-\bar{x}\right)}{\sigma^{2}\left(N-l\right)}$$

Where $\bar{x}$ represents the mean of the time series, $\sigma^{2}$ is its variance, and *l* is the lag. This equation ensures normalization, making the results comparable across different keypoints.

**Figure 11:**
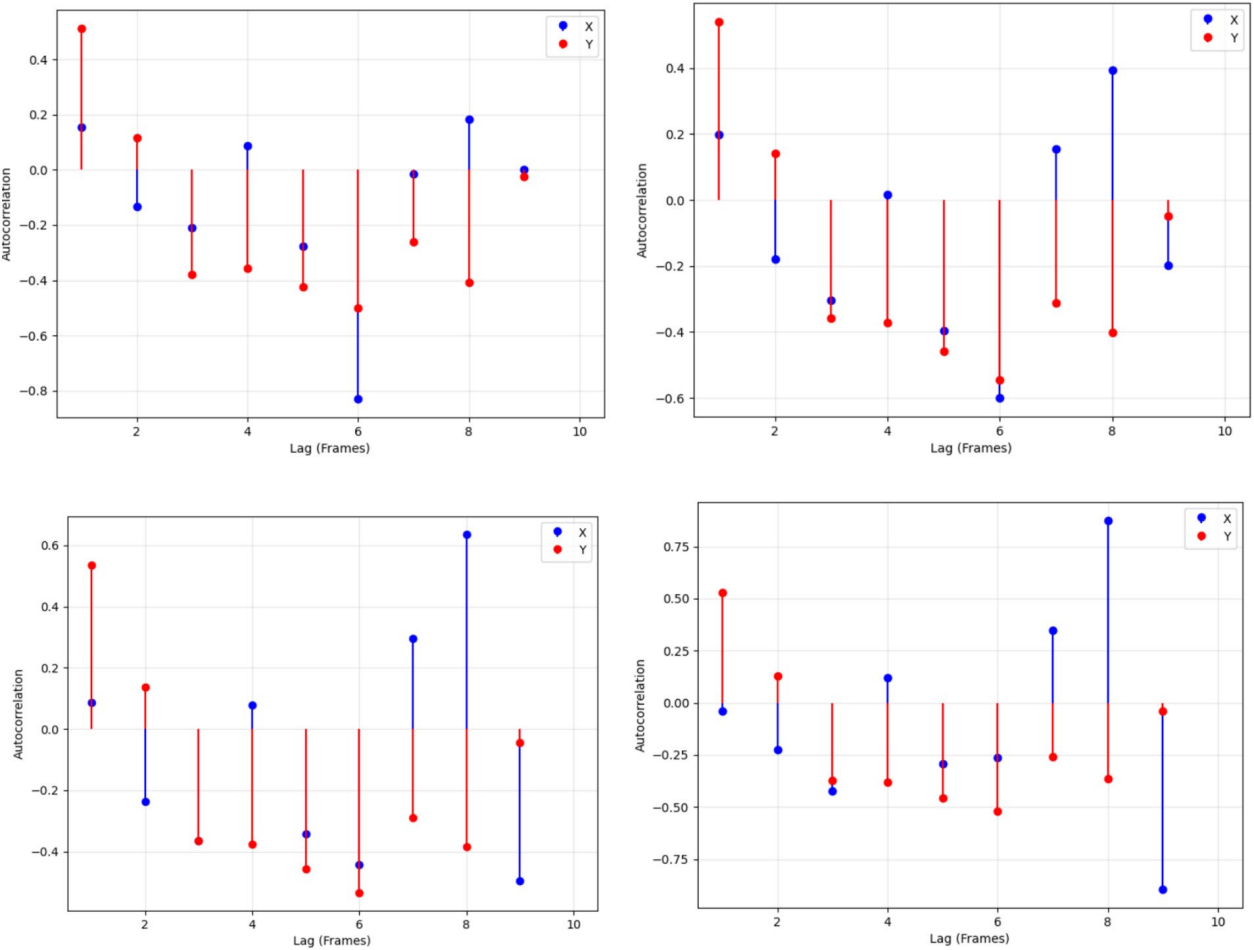
Autocorrelation plots of keypoint trajectories showing temporal dependencies in *x-*(blue) and *y-*coordinates (red) across different lags.

The MediaPipe Pose model pulled keypoint motion data from several frames in sequence. The 33 keypoint tracks from the videos produce x-axis and y-axis measurement data at each point. The ACF analysis for each important body position proceeded up to 10 frame delays. This technique uses data points to determine how movement at one spot aligns with changes at different points later on to find usual movement patterns over time. The resulting ACF results appeared next to each keypoint to show how human movements evolve over time. A sequence of regular movements generates distinct autocorrelation patterns, whereas irregular motions remain less predictable in the analysis. This feature representation effectively captures both local and global movement patterns, enhancing the recognition of complex human actions based on their temporal behavior. In Figure 11 the autocorrelation plots for keypoint motion trajectories are shown, illustrating temporal dependencies in both *x-* and *y-*coordinates for each keypoint.

### 3.7 Feature optimization

The accuracy of our classification model improves when extracted features are optimized. Due to its efficiency optimization (Herrera-Alcántara, 2022; Ye and Du, 2021), the gradient descent algorithm serves as an effective optimization tool, minimizing loss values and achieving stable results. The gradient descent algorithm changes the model parameter weights multiple times toward a loss function minimum. We define our loss function as Mean Squared Error (MSE) which measures the size of prediction errors in our study shown in Equation 23:


(23) $$ℒ=\frac{1}{N}\overset{N}{\underset{i=1}{\sum }}{\left(y_{i}-{\hat{y}}_{i}\right)}^{2}$$

Here $y_{i}$ represents the actual value, ${\hat{y}}_{i}$ is the predicted value, and *N* is the total number of samples. The algorithm calculates how each model weight affects loss and uses this information to update the weights in the Equation 24:


(24) $$\omega_{t+1}=\omega_{t}-\eta \nabla ℒ$$

The equation uses $\omega_{t}$ at the time *t* where η controls learning speed and ∇ℒ measures loss function derivatives. The learning rate supports the algorithm by defining its process steps while minimizing the risks of moving too quickly.

We applied the gradient descent approach to refine the extracted features and optimize them for classification. This algorithm iteratively adjusts model parameters to minimize the squared prediction errors. Our experiments set *η* at the best possible rate between stability and performance then stopped training when loss stopped improving. Experimental testing determined the appropriate learning rate value (η) from different testing conditions. The experimental procedure utilized values between 0.0001 and 0.01 to determine a learning rate which achieved stability together with minimum loss performance. An η value exceeding 0.005 typically led to system instability which caused MSE loss to oscillate or diverge. The training time became longer when using learning rates smaller than *η* = 0.0005 even though accuracy did not improve.

**Figure 12:**
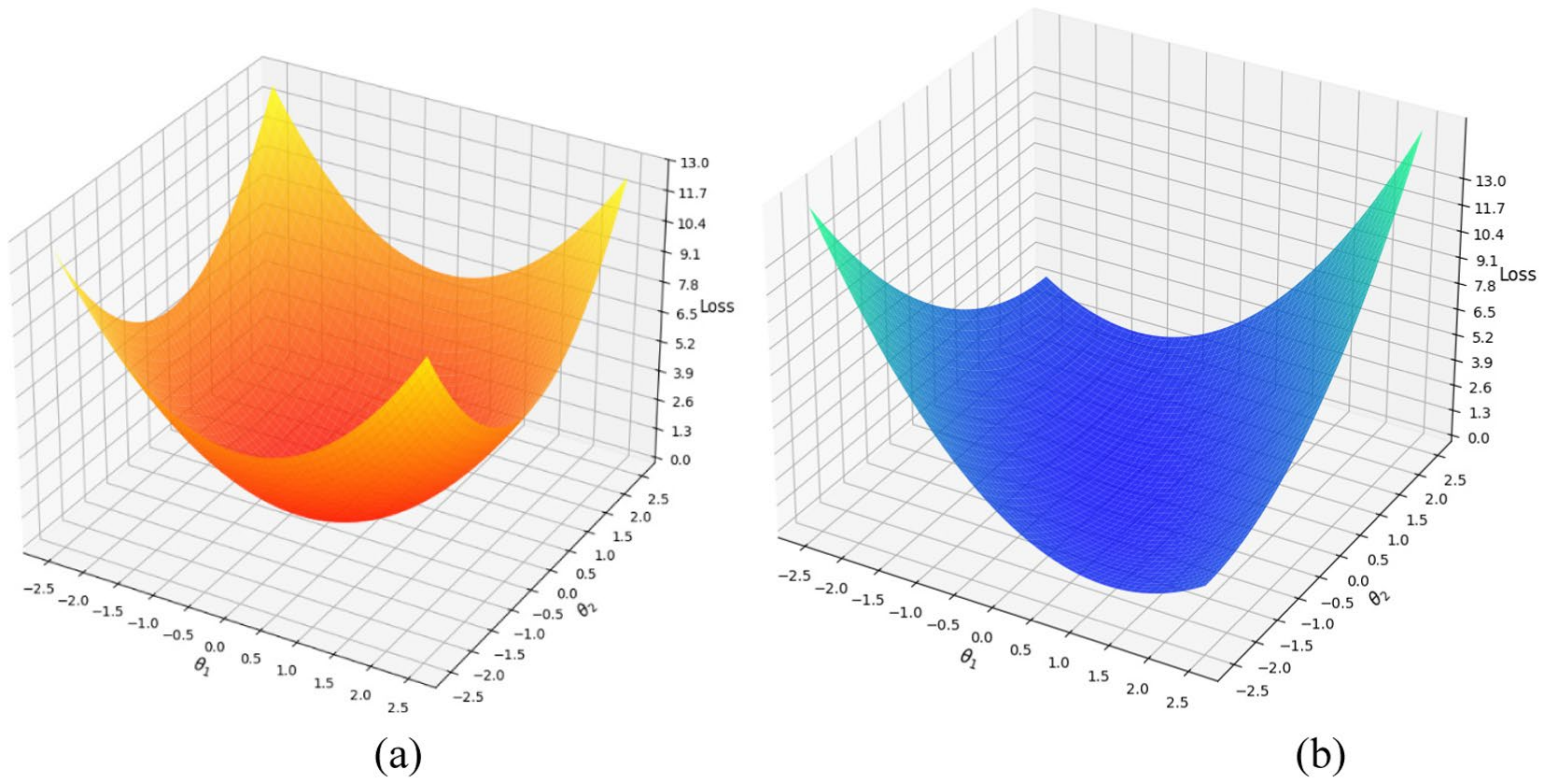
Plots showing the gradient descent optimization process on two datasets: **(a)** MOD20, and **(b)** Okutama-Action dataset.

The selected learning rate for this process proved to be *η* = 0.001 through comprehensive assessment. The chosen value helped the gradient descent optimizer to reach stable convergence while efficiently reducing the MSE loss. This optimization ensures that the refined features enhance action classification accuracy and improve overall performance in human action recognition. Gradient descent connects feature extraction and classification features to boost overall system performance. Figure 12 shows the plot of gradient descent optimizer on two datasets.

### 3.8 Classification

In our system, the Convolutional Neural Network (CNN) outperformed Deep Belief Networks (DBN) and Recurrent Neural Networks (RNN) for multi-person action recognition. The ability of CNNs to discover hierarchical spatial features in input data makes them optimal for this application because they successfully extract relevant patterns from full-body feature and keypoint-based features. The CNN architecture (Azmat et al., 2023a,b) executes its operation on multiple layers which include convolutional and activation and pooling and fully connected layers (Hossain and Sajib, 2019). CNN performs the convolutional process as its fundamental operation with the following mathematical representation in Equation 25:


(25) $$z_{i,j}^{k}=\overset{M}{\underset{m=1}{\sum }}\overset{N}{\underset{n=1}{\sum }}x_{i+m-1,j+n-1}\;.\;\omega_{m,n}^{k}+b^{k}$$

Where $z_{i,j}^{k}$ is the activation value at position (*i, j*) in the *k*-th feature map, *x* represents the input patch, $\omega_{m,n}^{k}$ denotes the filter weights, and $b^{k}$ is the bias term. This operation enables the network to capture local spatial features.

The output from the last convolutional or fully connected layers passes through a SoftMax function to produce probabilities through logits transformation by using Equation 26:


(26) $$P\left(y=c|x\right)=\frac{\exp\left(z_{c}\right)}{\sum_{j=1}^{C}\exp\left(z_{j}\right)}$$

**Figure 13:**
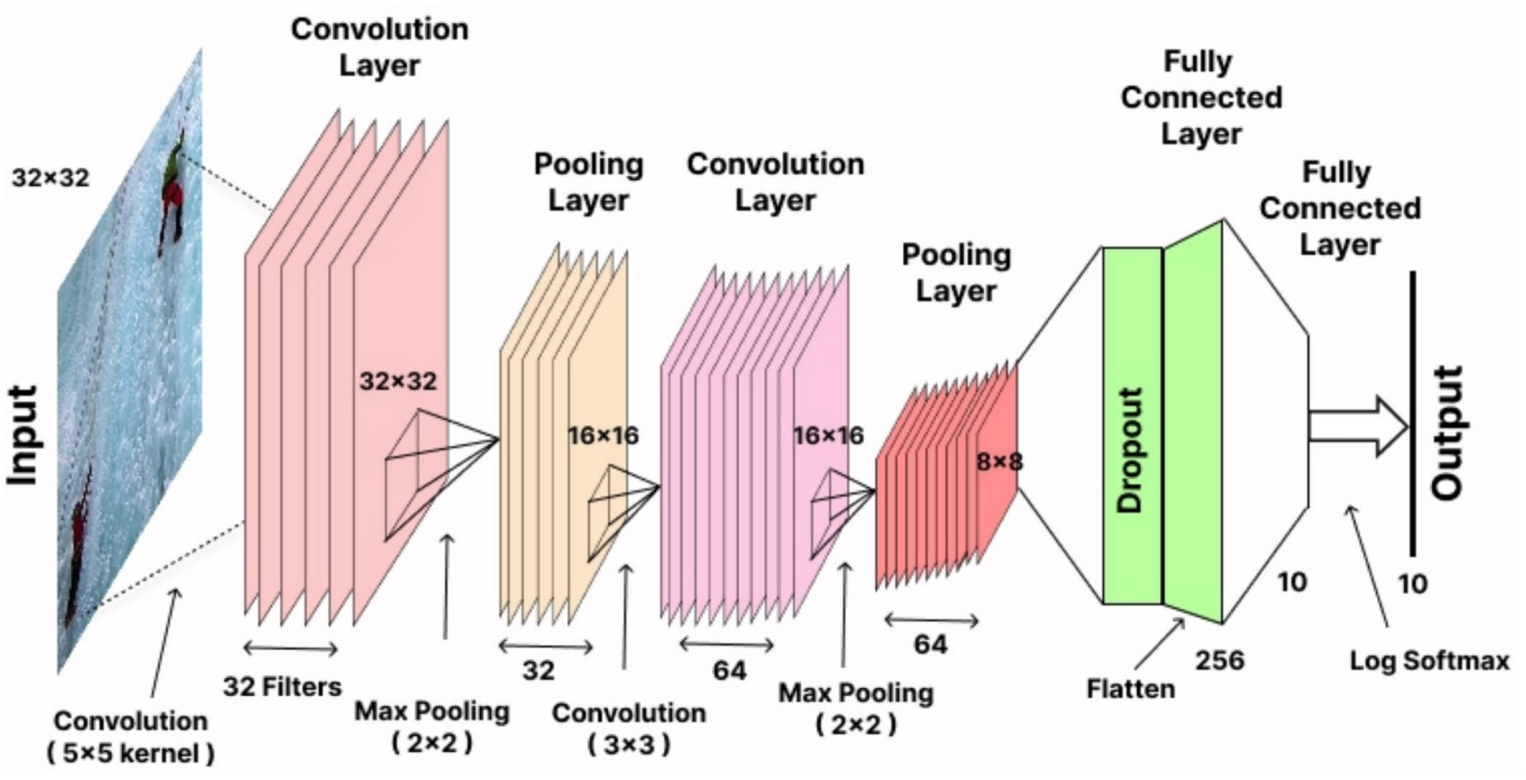
Convolutional Neural Network architecture for multi-person action recognition.

Where $P\left(y=c|x\right)$ is the probability of the input *x* belonging to class *c,* $z_{c}$ is the logit for class *c*, and *C* represents the total number of classes. This probabilistic output facilitates multi-class classification. During training CNN demonstrates automatic feature optimization capabilities as well as high classification precision which identifies it as the most efficient solution for human action recognition in our system. The experimental outcomes showed CNN achieved better results than DBN and RNN in terms of accuracy and precision and recall performance thereby validating its application in this domain. Figure 13 illustrates the detailed architecture of the CNN employed in the proposed system for multi-person action recognition.

## 4 Experimental setup and datasets

This section outlines the experimental setup, including dataset descriptions, system configuration, and evaluation metrics. A systematic assessment evaluates the effectiveness of UAV-based multi-person action recognition and compares its performance with existing approaches to ensure reliability. For the procedure, a Windows 10 PC with an Intel Core i7 processor running at 3.60 GHz, a Nvidia Tesla K80 with 2496 CUDA cores, and 16 GB of RAM was used. For both training and building the model, Python 3.6 and the Keras API were utilized.

The dataset was split into 80% training data along with 20% testing data for an accurate evaluation of model performance. Such data partition ensures the validation of proposed system performance accuracy across training data along with unseen testing data.

### 4.1 Datasets

For this study, we utilized two datasets: MOD20 and Okutama-Action. The details of each dataset are provided below.

#### 4.1.1 MOD20 dataset

**Figure 14:**
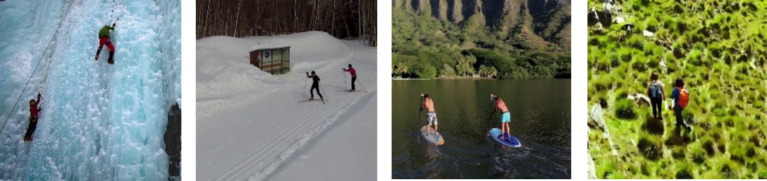
A few examples from the MOD20 dataset.

The MOD20 dataset (Perera et al., 2020) is a benchmark dataset specifically designed for human action recognition tasks in aerial imagery. This dataset encompasses contains videos captured by UAVs across multiple environmental conditions from different aerial viewpoints. This dataset contains 20 different action classes, in this study we selected six action classes: Rock climbing, Standup Paddling, Cycling, Skiing, Backpacking, and Running. These classes show multiple dynamic activities which contain changes in movement patterns as well as environmental changes and camera vantage points. The chosen dataset achieves appropriate representation of movements requiring accurate feature extraction and classification because it includes a range of activities. Thus, it provides sufficient evaluation for the proposed system’s effectiveness. Figure 14 depicts a few examples of the MOD20 dataset.

#### 4.1.2 Okutama-Action dataset

**Figure 15:**
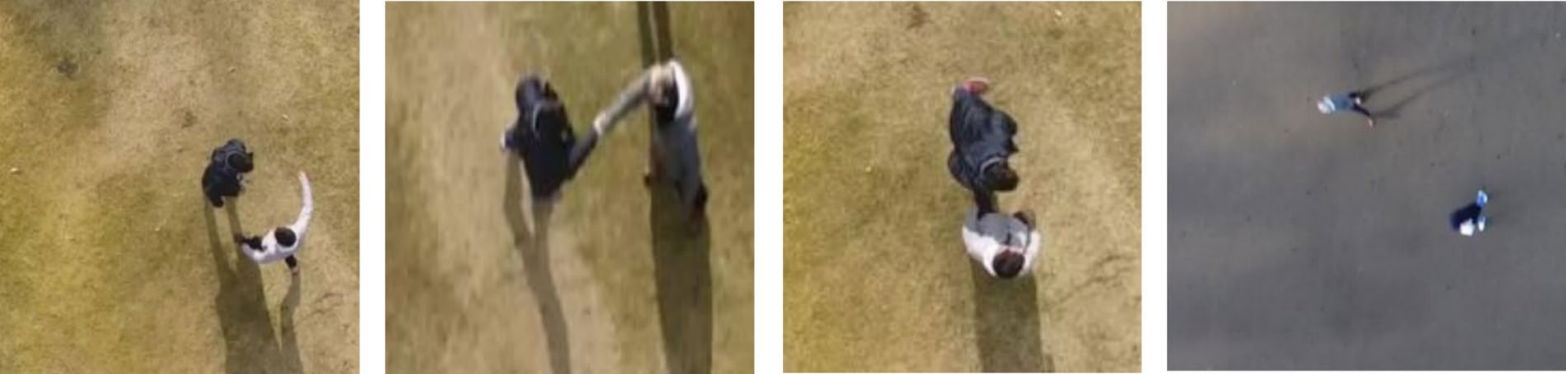
A few examples from the Okutama-Action dataset.

The Okutama-Action dataset (Marti et al., 2017) employed incorporates seven distinct actions which include Carrying, Handshaking, Hugging, Pushing, Sitting, Running, and Walking. The dataset shows human actions performed in numerous outdoor situations using UAV cameras which makes it hard to recognize human movements because of scaling variations and changing viewpoints. The complexity of the dataset proves the strength of the proposed system to detect human activities dynamically. Figure 15 illustrates some images from the Okutama-Action dataset.

### 4.2 Results and analysis

In this section, we performed several kinds of experiments to determine the accuracy of the proposed model’s classification across benchmark datasets. The aim was to verify its effectiveness by comparing it with other state-of-the-art methods.

#### 4.2.1 Confusion matrix

This section evaluates the proposed system when processing two benchmark datasets namely MOD20 with Okutama-Action. The system classification accuracy appears in Tables 1, 2 through confusion matrix representations of the analysis results from both datasets. UAV imagery delivers an effective system through matrices that record true positive and negative results and spurious outputs for each class category.

**Table 1:** Confusion matrix for multi-person action recognition accuracy over MOD20 dataset.

Classes	Rock climbing	Standup paddling	Cycling	Skiing	Backpacking	Running
Rock climbing	92	3	2	1	1	1
Standup paddling	2	91	3	2	1	1
Cycling	3	2	92	1	1	1
Skiing	1	3	1	91	3	1
Backpacking	2	1	2	3	91	1
Running	1	2	1	3	1	92
**Mean accuracy = 91.50%**						

Bold value in the last row indicates the mean accuracy across all classes.

**Table 2:** Confusion matrix for multi-person action recognition accuracy over Okutama-Action dataset.

Classes	Carrying	Handshaking	Hugging	Pushing	Sitting	Running	Walking
Carrying	89	2	3	1	2	1	2
Handshaking	1	90	2	3	1	2	1
Hugging	1	1	90	2	3	1	2
Pushing	2	1	1	89	3	2	2
Sitting	2	2	1	2	91	1	1
Running	1	2	1	1	1	90	4
Walking	2	3	1	3	1	1	89
**Mean accuracy = 89.71%**							

Bold value in the last row indicates the mean accuracy across all classes.

#### 4.2.2 Precision, recall, and F1-score evaluation

The proposed system’s performance evaluation section shows assessment results based on precision alongside recall as well as F1-score metrics on each benchmark dataset. The precision rate describes how many correct positive predictions exist among all positive predictions made by the system while recall shows the proportion of correctly identified positives versus total actual positives and the F1-score represents their harmonic mean.

The presented data in Tables 3, 4 provide precision and recall scores with F1-scores of various datasets including MOD20 and Okutama-Action. The system performs in a reliable manner due to its consistent operational capacity within environments with various action types.

**Table 3:** The overall accuracy, precision, recall, and F1 score over the MOD20 dataset.

Classes	Precision	Recall	F1 Score
Rock climbing	0.91	0.92	0.92
Standup paddling	0.89	0.91	0.90
Cycling	0.91	0.92	0.92
Skiing	0.90	0.91	0.91
Backpacking	0.93	0.91	0.92
Running	0.95	0.92	0.93
**Mean**	**0.915**	**0.915**	**0.917**

Bold value in the last row represents the mean values for each metric across all classes.

**Table 4:** The overall accuracy, precision, recall, and F1 score over the Okutama-Action dataset.

Classes	Precision	Recall	F1 Score
Carrying	0.91	0.89	0.90
Handshaking	0.89	0.90	0.90
Hugging	0.91	0.90	0.90
Pushing	0.88	0.89	0.89
Sitting	0.89	0.91	0.90
Running	0.92	0.90	0.91
Walking	0.88	0.89	0.89
**Mean**	**0.897**	**0.897**	**0.899**

Bold value in the last row represents the mean values for each metric across all classes.

#### 4.2.3 Comparison with existing methods

The evaluation of system effectiveness involved performing classification accuracy comparison against multiple state-of-the-art techniques. Table 5 demonstrates an inclusive accuracy comparison of the analyzed approaches through benchmarks from this study.

**Table 5:** Comparison of multi-person action recognition accuracies over MOD20 and Okutama-Action datasets.

Method	MOD20	Okutama-Action
Perera et al. (2020)	66.50	–
Perera et al. (2020)	74.0	–
Vrskova et al. (2023)	78.21	–
Dhiman et al. (2024)	86.13	–
Algamdi et al. (2022)	–	47.50
Khan et al. (2024)	–	60.76
Ahmad et al. (2022)	–	75.4
Yang et al. (2019)	–	85.2
**Proposed**	**91.50**	**89.71**

Bold value in the last row signifies the performance of the proposed system compared to other methods.

The proposed system delivers better results than all existing methods in benchmark datasets which proves both its reliable performance and improved abilities in recognizing human actions. The Table 6 highlights the significant accuracy achieved by our proposed method due to its advanced feature extraction techniques, gradient descent optimization, and CNN-based classification.

**Table 6:** Ablation study of pipeline components on MOD20 and Okutama-Action datasets across classifier.

Experiment	Preprocessing	Segmentation (GMM)	Keypoint extraction	Full-body features	Keypoint based features	Gradient descent optimizer	Classifiers			Datasets	
							CNN	RNN	DBN	MOD20 (%)	Okutama-Action (%)
Baseline	✓	x	x	x	x	x	x	x	✓	40.27	39.76
Baseline	✓	x	x	x	x	x	x	✓	x	41. 42	40.66
Baseline	✓	x	x	x	x	x	✓	x	x	61.72	49.88
Preprocessing	x	✓	x	✓	x	✓	x	x	✓	46.10	42.09
Preprocessing	x	✓	x	✓	x	✓	x	✓	x	44. 44	42.72
Preprocessing	x	✓	x	✓	x	✓	✓	x	x	63.52	52.86
Segmentation (GMM)	✓	x	✓	x	✓	✓	x	x	✓	47.60	45.01
Segmentation (GMM)	✓	x	✓	x	✓	✓	x	✓	x	49. 14	46.72
Segmentation (GMM)	✓	x	✓	x	✓	✓	✓	x	x	65.21	55.16
Keypoint extraction	✓	✓	x	✓	x	✓	x	x	✓	50.63	49.91
Keypoint extraction	✓	✓	x	✓	x	✓	x	✓	x	50. 59	52.48
Keypoint extraction	✓	✓	x	✓	x	✓	✓	x	x	69.07	57.66
Full-body features	✓	✓	✓	x	✓	✓	x	x	✓	53.97	50.23
Full-body features	✓	✓	✓	x	✓	✓	x	✓	x	54. 09	56.39
Full-body features	✓	✓	✓	x	✓	✓	✓	x	x	72.14	60.35
Keypoint based features	✓	✓	✓	✓	x	✓	x	x	✓	59.27	55.09
Keypoint based features	✓	✓	✓	✓	x	✓	x	✓	x	65. 40	62.44
Keypoint based features	✓	✓	✓	✓	x	✓	✓	x	x	79.31	66.24
Gradient descent optimizer	✓	✓	✓	✓	✓	x	x	x	✓	70.67	69.39
Gradient descent optimizer	✓	✓	✓	✓	✓	x	x	✓	x	73. 54	70.07
Gradient descent optimizer	✓	✓	✓	✓	✓	x	✓	x	x	85. 01	80.22
Proposed system	✓	✓	✓	✓	✓	✓	x	x	✓	87. 17	83.29
Proposed system	✓	✓	✓	✓	✓	✓	x	✓	x	89. 33	85.57
Proposed system	✓	✓	✓	✓	✓	✓	✓	x	x	91. 50	89.71

Colors associated with the tick (✓) and cross (x) symbols are used to visually distinguish between the methods applied and not applied in experiment. The red cross (x) indicates that a particular technique is not used in the experiment, while the orange tick (✓) signifies that the method is included. This color coding helps to quickly differentiate between the applied and non-applied techniques across the various experiments.

## 5 Discussion

The system’s ability to recognize human actions using UAV imagery is validated through experimental results. The system produced excellent results across all datasets that were evaluated, particularly when CNN was used for classification tasks. CNN consistently achieved the best performance results among the various classifiers, achieving the maximum level of accuracy across all test data sets. This discovery is consistent with CNN’s well-known ability to learn high-level features and determine spatial hierarchies from complex datasets.

CNN’s ability to recognize and extract spatial patterns in UAV footage with varying perspective angles, size variations, and unique surroundings is the key to its effectiveness in image categorization. RNN’s accuracy performance was lower when used for static feature-based tasks. When processing complex spatial interactions, CNN showed a greater degree of adaptation than DBN, but the results were still good. Classifier performance study shows why model selection needs to be done according to application requirements and dataset characteristics. CNN classifier integration is a crucial component of the system that maximizes accuracy and improves performance efficiency. Important information required for UAV-based action recognition is revealed by classifier selection techniques. Furthermore, the comparison shows that the suggested approach sets higher benchmarks for classification accuracy and UAV-based human action identification features.

### 5.1 Real-world challenges in UAV-captured videos

The proposed system shows outstanding results on benchmark datasets however it must tackle challenges that occur in real-world UAV-based operations. The view of obstacles including buildings or trees with other environmental elements causes reduced visibility which leads to possible errors in detection outcomes. The pose estimation techniques utilized by the system help it maintain stability against these obstacles because they detect skeletal keypoints instead of full-body silhouettes. The system successfully detects human poses through tracking visible keypoints including shoulders, elbows, and knees despite partial body obstructions.

Extreme occlusions and crowded surroundings are still obstacles. Future research will address these by utilizing improved occlusion-handling techniques with predictive modeling, incorporating scene segmentation approaches to differentiate barriers from target humans, and integrating temporal information from sequence video frames to recover missing keypoints. Together with its excellent benchmark performance, these improvements will guarantee the systems resilience and flexibility to real-world scenarios.

### 5.2 Robustness to real-world scenarios

The proposed system has been evaluated on benchmark datasets that cover a variety of conditions with different perspectives, scales, and environmental elements, MOD20 and Okutama-Action. These datasets provide a reliable framework for evaluating the systems functionality under challenging conditions.

To address lighting variations, preprocessing techniques such as Gaussian blur and grayscale conversion are applied to improve image quality in different lighting conditions. These preprocessing steps ensure that the input data remains consistent, even when captured under different lighting conditions.

Also, the system is robust against occlusions by utilizing pose estimation techniques that identify skeleton keypoints. By capturing invariant information, feature extraction techniques like motion-based histograms and Fourier descriptors allow the system to remain accurate even when perspective shifts and dynamic movements occur. These combined methods show that the system can adapt to difficult situations, as shown by its high accuracy on all benchmark datasets.

### 5.3 Real-time feasibility and future deployment

The proposed framework’s performance has been validated under a variety of controlled circumstances by evaluating it on pre-recorded benchmark datasets, including MOD20 and Okutama-Action. These datasets offer a solid basis for evaluating the precision, resilience, and computational effectiveness of the system.

While there has not been any real-time testing in a UAV context yet, the computational complexity analysis shows that the framework is callable and computational efficient. While CNN-based classification achieves efficient processing appropriate for real-time applications, preprocessing and feature extraction techniques are tuned to minimize overhead.

Future work will evaluate the system’s real-time feasibility under hardware limitation such as processor power and energy consumption by implementing it on UAV hardware. To guarantee compatibility with embedded system frequently found in UAVs, hardware-specific optimizations will be investigated, improving the framework’s suitability for practical situations.

## 6 Computational complexity analysis

The computational complexity of the proposed system was analyzed for each stage, as shown in the Table 7.

**Table 7:** Computational complexity of the proposed system.

Stage	Operation	Complexity
Preprocessing	Gaussian Blur	*O(n)*
Segmentation	GMM Segmentation	*O(n)*
Feature extraction	AKAZE	*O(n)*
Feature extraction	Fourier Descriptor	*O(n* logn *)*
Feature extraction	Distance Transform	*O(n)*
Feature extraction	0–180° intensity	*O(n* logn *)*
Feature extraction	Keypoint-based motion histogram	*O(n)*
Feature extraction	Multi-point autocorrelation	*O(* n *)*
Optimization	Gradient Descent	*O(n* logn *)*
Classification	CNN	*O(n* logn *)*

## 7 Performance of different classifiers

Our system included three distinct classifiers: recurrent neural networks (RNN), deep belief networks (DBN), and convolutional neural networks (CNN). The ability of the classifiers to identify human action from UAV images across all benchmark datasets was the main focus of their evaluation. CNN analyzed all features and every detail across all benchmark datasets, it showed remarkable performance. While processing UAV data, the convolutional layers collected crucial feature data that proved challenging to manage due to changes in image scale as well as disparate sizes and perspectives. The accuracy of CNN models and DBN’s benchmark performance were near. The spatial variety found in UAV datasets is not adequately fitted by the layer-wise pretraining of DBN. Convolutional neural networks (CNN), deep belief networks (DBN), and recurrent neural networks (RNN) were the three classification techniques we used to test our system and evaluate its efficacy. The classifiers were evaluated on how well they were able to recognize human actions from UAV footage across all benchmark datasets. Because CNN efficiently extracted spatial information while identifying intricate details within the images, it produced the best results across all datasets. Even when processing UAV data, the model’s convolutional layers learned significant feature representations, which presented challenges because of shifting resolutions, sizes, and perspectives. As a benchmark model the DBN maintained a slight disadvantage in accuracy against CNN models. Its layer-wise pretraining method fails to adapt properly to diverse spatial features because it exists in UAV datasets. Despite its restrictions DBN demonstrated reliable performance which makes it suitable for utilization in systems requiring quick computation.

Our system achieved moderate results with the RNN because of its competency in processing sequential information which depends on time ordering. Due to the emphasis on static feature-based tasks in the system framework the RNN failed to optimally engage with temporal relations which led to reduced performance accuracy. Application success depends on using classifiers that match both extracted features along with their necessary specifications. The RNN structure works best on sequences but it showed limitations during UAV static image processing as part of this study. Research findings confirm that RNN maintains its use as an effective methodology for tasks that require recognizing temporal dependencies during classification. The results are presented in Table 8 to show the evaluation outcomes of each model for benchmark dataset classification accuracy. The results demonstrate that CNN serves as the best choice for UAV-based human action recognition because it shows exceptional capability in extracting and generalizing complex features.

**Table 8:** Classification accuracy of different classifiers across datasets.

Datasets	CNN%	RNN%	DBN%
MOD20	91.50	89.33	87.17
Okutama-Action	89.71	85.57	83.29

**Figure 16:**
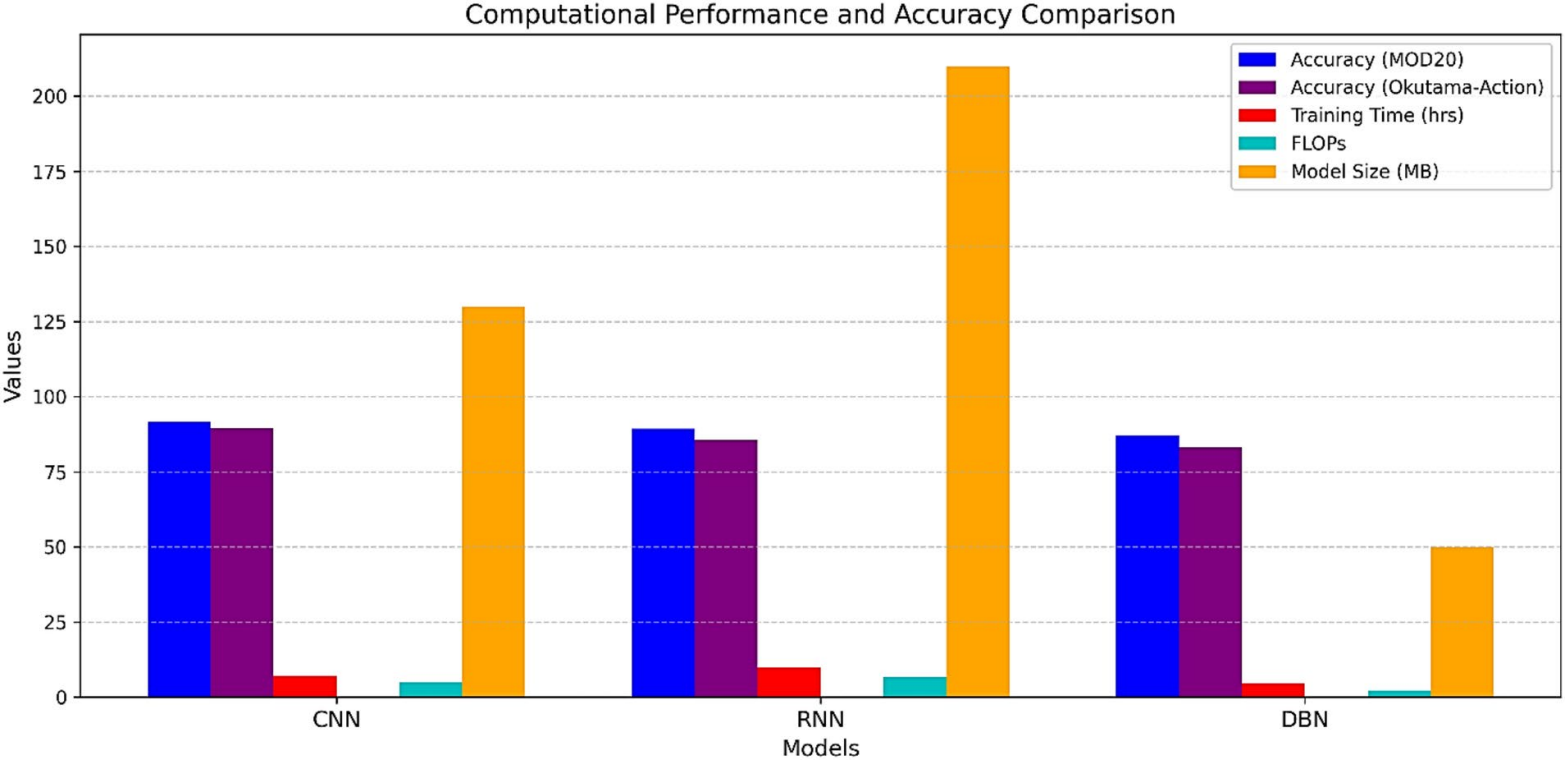
Comparison of training time, inference time, FLOPS, model size, and accuracy across classifiers.

A performance and efficiency analysis of the proposed system included DBN, CNN and RNN classifiers. Figure 16 presents the main metrics including training time, inference time, FLOPs, model size and accuracy. The study demonstrates that CNN delivers maximum accuracy of 91.50% thus it functions as the best classification approach for this proposed system.

## 8 Conclusion

This study demonstrated the effectiveness of a comprehensive system for multi-person action recognition utilizing UAV imaging on several benchmark datasets, such as MOD20, and Okutama-Action. The system integrates sophisticated preprocessing with feature extraction approaches and deep learning classifiers to deliver accurate results. The CNN classifier achieved superior performance compared to its counterparts DBN and RNN since it demonstrated effectiveness in extracting spatial features from UAV imagery while handling according to changes in perspective and scale. The system achieves reliable status as a UAV-based human action recognition solution through these testing outcomes which also demonstrate its robust functionality.

Future work will focus on resolving the problem of occluded human actions since this issue persists in current system implementations. The real-world operations of UAV systems frequently encounter occlusions because they fly through dense surroundings and partially restricted viewpoints. Our future system development will integrate advanced methods to identify hidden human figures through the combination of time-related insights and situational context information. The system becomes more robust when this enhancement takes effect thus enabling broader application in dynamic complex situations.

## Data Availability

Publicly available datasets were analyzed in this study. This data can be found here: https://asankagp.github.io/mod20/; https://paperswithcode.com/dataset/okutama-action.
